# UBIAD1 and CoQ10 protect melanoma cells from lipid peroxidation-mediated cell death

**DOI:** 10.1016/j.redox.2022.102272

**Published:** 2022-02-18

**Authors:** Liaisan Arslanbaeva, Giovanni Tosi, Marco Ravazzolo, Manuela Simonato, Francesco A. Tucci, Salvatore Pece, Paola Cogo, Massimo M. Santoro

**Affiliations:** a Laboratory of Angiogenesis and Cancer Metabolism, DiBio, University of Padua, Italy; b Fondazione Istituto di Ricerca Pediatrica "Città della Speranza", Padova, Italy; c IEO, European Institute of Oncology IRCCS, Milan, Italy; d Division of Pediatrics, Department of Medicine, University Hospital S Maria della Misericordia, University of Udine, Italy; e Veneto Institute of Molecular Medicine (VIMM), Padua, Italy

**Keywords:** UBIAD1, Lipid peroxidation, CoQ10, NQO1, Antioxidant response, Melanoma

## Abstract

Cutaneous melanoma is the deadliest type of skin cancer, although it accounts for a minority of all skin cancers. Oxidative stress is involved in all stages of melanomagenesis and cutaneous melanoma can sustain a much higher load of Reactive Oxygen Species (ROS) than normal tissues. Melanoma cells exploit specific antioxidant machinery to support redox homeostasis. The enzyme UBIA prenyltransferase domain-containing protein 1 (UBIAD1) is responsible for the biosynthesis of non-mitochondrial CoQ10 and plays an important role as antioxidant enzyme. Whether UBIAD1 is involved in melanoma progression has not been addressed, yet. Here, we provide evidence that UBIAD1 expression is associated with poor overall survival (OS) in human melanoma patients. Furthermore, UBIAD1 and CoQ10 levels are upregulated in melanoma cells with respect to melanocytes. We show that UBIAD1 and plasma membrane CoQ10 sustain melanoma cell survival and proliferation by preventing lipid peroxidation and cell death. Additionally, we show that the NAD(P)H Quinone Dehydrogenase 1 (NQO1), responsible for the 2-electron reduction of CoQ10 on plasma membranes, acts downstream of UBIAD1 to support melanoma survival. By showing that the CoQ10-producing enzyme UBIAD1 counteracts oxidative stress and lipid peroxidation events in cutaneous melanoma, this work may open to new therapeutic investigations based on UBIAD1/CoQ10 loss to cure melanoma.

## Highlights

• UBIAD1 is associated with poor overall survival (OS) of melanoma patients and is highly expressed in melanoma cell lines.
• Melanoma cell lines are dependent on UBIAD1 and plasma membrane CoQ10 for cell survival.
• UBIAD1 and COQ10 protect melanoma cells from lipid peroxidation and apoptotic cell death.
• NQO1 is required for UBIAD1/CoQ10-mediated melanoma lipid peroxidation protection and cell survival.

## 1 Introduction

Although accounting for only 5% of all skin cancers, cutaneous melanoma represents the deadliest form of skin cancer with the highest levels of mutational load [1]. Melanoma cells have elevated ROS levels and elevated antioxidant defense compared to melanocytes, in which oxidative stress is already close to a survival threshold due to increased metabolism, melanin biosynthesis and ultraviolet radiation [2]. Melanoma tumors have evolved redox adaptive mechanisms to counteract oxidative stress, such as the activation of the nuclear factor erythroid 2–related factor 2 (NRF2), the upregulation of metabolic survival pathways, including serine biosynthesis and the pentose phosphate pathway, and the elevation of redox metabolites such as glutathione (GSH) and nicotinamide adenine dinucleotide phosphate (NADPH) [3]. The increase of ROS levels by giving oxidant treatments or by removing cellular antioxidant systems could serve as a therapeutic approach to trigger cell death in melanomagenesis and melanoma progression [4,5].

It has been recently suggested that the induction of lipid peroxidation (LP) could serve as a special target in melanomagenesis. Several protective mechanisms against lipid peroxidation in melanoma cells have been described by different studies [6]. For example, oleic acid from lymph, which is incorporated in plasma membrane phospholipids, was found to protect metastatic melanoma cells from lipid peroxidation [7]. Also, a striking upregulation of Sterol Regulatory Element-Binding Protein 2 (SREBP2), a lipogenesis regulator, and a decrease of lipid peroxidation levels have been found in cultured and in circulating single melanoma cells freshly isolated from blood specimens [8]. A similar role in protection against lipid peroxidation in BRAF-inhibitor resistant melanoma was shown for SREBP1, another master regulator of lipogenesis [9]. Another study [10] highlighted the role of aldo-keto reductases in melanoma survival, which convert aldehydes and ketones to their corresponding alcohols, which are in turn able to detoxify lipid peroxides and thus to inhibit cell death execution.

Therefore, increasing evidence indicates that tumor cells have evolved several defense mechanisms to suppress lipid peroxidation in parallel to the well-known defense mechanism mediated by GPx4 [11]. One of these newly discovered strategies is through the enzyme-mediated reduction of the plasma membrane CoQ10 [12]. It has been shown that ferroptosis suppressor protein 1 (FSP1, also called AIFM2) functions as an oxidoreductase to reduce ubiquinone (CoQ10) to ubiquinol (CoQ10H_2_) mainly on the plasma membrane [11,13] Another very recent study published that dihydroorotate dehydrogenase (DHODH), located in the mitochondrial inner membrane, is important for the reduction of CoQ10 [14]. Along with these two enzymes, also NQO1 could play a significant role in CoQ10 reduction on the plasma membrane of melanoma cells, since it was published that NQO1 is important for melanoma survival [15]. Finally, it is important to mention that most current studies published that lipid peroxidation in cancer cells induces ferroptosis-dependent cell death, a newly described form of iron-dependent, regulated non-apoptotic cell death [16]. Overall, CoQ10 is an essential antioxidant involved in both mitochondrial bioenergetics and plasma membrane protection [17]. Indeed, CoQ10 protects membranes against oxidative damage directly by disrupting the lipid peroxidation chain and through maintaining the plasma membrane redox system [13,[18], [19], [20]].

UBIAD1 is a novel transmembrane enzyme localized in the Golgi apparatus and the endoplasmic reticulum (ER) and it is responsible for the biosynthesis of plasma membrane CoQ10 [21,22]. Here we show for the first time that UBIAD1 plays an antioxidant tumor-promoting role in melanoma cells by supporting plasma membrane CoQ10 synthesis and, thus, contributing to protect membrane components against lipid peroxidation and cell death.

## 2 Materials and methods

### 2.1 Cell culture

Melanoma cell lines SkMel3, Mel Juso and IPC298 were given by Dr. Martin Bergo (Karolinska Institute, Sweden); 1205Lu, WM88, WM164, WM1366, WM3734a by Dr. Ivan Bogeski (University of Goettingen, Germany); SkMel28, A375, MM052 and MM165 by Dr. Luca Scorrano (Univerisity of Padua, Italy); SkMel24 and RPMI-7951 by Dr. Marina De Bernard (University of Padua, Italy); normal human epidermal melanocytes NHEM (ATCC #PCS-200-012) by Dr. Barbara Stecca (ISPRO, Italy). Melanoma cell lines and HEK293T virus-packaging cell lines were maintained in DMEM high glucose GlutaMAX™ medium (Gibco) containing 10% FBS and 100 μg/ml penicillin and 100 μg/mL streptomycin. Human Epidermal Melanocytes HEMa-LP (#C-024-5C) were purchased from Invitrogen and maintained in Medium 254 (Gibco) containing HMGS-2 (Gibco), 10% FBS and 100 μg/ml penicillin and 100 μg/mL streptomycin. All cell lines were free of mycoplasma contamination. All cell lines were cultured in a humidified incubator at 37°C with 5% CO2.

Cell treatments were performed on melanoma cell lines SkMel28, A375 and Mel Juso plated in 6-well plates and allowed to adhere overnight. 1 μM of staurosporine (#569396, Sigma), 1 μM of RSL3 (#SML2234, Sigma), 5 μM of FIN56 (#1740, Sigma), 10 μM of Erastin (#E7781, Sigma), 100 μM of cumene hydroxyperoxide (CH) (component F, #C10446, ThermoFisher) were supplemented to the original medium. Treatment with an equal amount of vehicle (DMSO) was used to assess 100% viability. Samples were collected after 16 h and analyzed by Western blotting as described below.

### 2.2 Lentiviral production and UBIAD1 or NQO1 silencing in melanoma cells

Lentiviruses encoding shRNA-UBIAD1 (5'-CCGGTGTCGGGAGAGACTGTCAAAGCTCGAGCTTTGACAGTCTCTCCCGACATTTTTG-3') and shRNA-NQO1 (5'-TGGAAGAAACGCCTGGAGAAT-3') were produced in HEK293T cells with packaging vectors pMD2.G (VSV-G envelope), pMDLg/pRRE (Gag/Pol), and pRSV-Rev (Rev) by polyethylenimine (PEI)-mediated transfection. Virus production was performed following this protocol: 56.7 μL of 1 mG/mL of PEI was diluted in 500 μL of Opti-MEM GlutaMAX™ media (Gibco) per 10 cm plate then 18.9 μG of DNA mix (7.28 μG of Gag/Pol, 2.91 μG of Rev, 1.46 μG of VSV-G, 7.28 μG of experimental plasmid pLKO or pLKO-shRNA-UBIAD1) was diluted in 500 μL of Opti-MEM GlutaMAX™ media (Gibco) per 10 cm plate, next DNA/PEI mixtures were incubated at room temperature for 20 min, then complexes were added drop-wise to cells. Fresh medium was replaced after 18 h. Lentivirus-containing supernatant was harvested 48 and 96 h later, passed through 0.45 μm syringe filters (Starlab), collected by ultracentrifugation at 31,900 rpm 2 h 4°C and resuspended in 1× PBS. Virus aliquots were stored at −80°C. To induce knockdown of UBIAD1 and NQO1, cells were transduced with lentiviral supernatants in 6-well plates at 1×10^5^/well. At time of plating, cells were transduced with suspended virus particles in the presence of 0.5 μg/mL of polybrene (Sigma). Fresh media was replaced after 24 h.

Cells were infected with lentiviruses containing pLKO empty plasmid (named as control) or pLKO plasmid containing shRNA-UBIAD1 or shRNA-NQO1. For most experiments, where not stated otherwise, the two genes were strongly silenced (named as UBIAD1^KD high^ or simply UBIAD1^KD^ and NQO1^KD high^ or simply NQO1^KD^). A lower level of silencing was instead used for co-silencing and dose-dependent experiments (referred to as UBIAD1^KD low^ and NQO1^KD low^). The relative level of silencing was validated by qRT-PCR (Fig. S4F).

### 2.3 Cell proliferation assay

1.5×10^3^ SkMel28 and Mel Juso and 2×10^3^ A375 cells/well were plated in 96-well plates in standard media and allowed to adhere 6 h before proceed with lentivirus transduction. Fresh media was replaced after 16 h and after 1 and 3 days of lentiviral infection. Every day, during the next 5 days from transduction, one plate was washed 3 times with 1× PBS, fixed in 3.7% paraformaldehyde for 10 min at room temperature (RT) and incubated in 0.1% of crystal violet solution in deionized water with gentle shaking. The plate was then washed 4 times with tap water and air-dried at RT for at least 24 h. Then 200 μL of 100% of methanol was added to each well and gently shaken on the rotator for 20 min to dissolve the crystal violet. The absorbance of each well was measured at a wavelength of 560 nm with a plate reader (Tecan).

### 2.4 Idebenone rescue experiments

Idebenone rescue was analyzed by growth curve assay of proliferation, by Western blotting assay of proliferation markers (RRM2 and cyclin A) and by lipid peroxidation FACS assay. Idebenone-dependent rescue analyses were performed on 96-well plates. 1.5×10^3^ SkMel28 and Mel Juso and 2×10^3^ A375 cells/well were plated in standard media and allowed to adhere 6 h. Concentration of idebenone (Tocris) was varied according to cell line: 1 μM idebenone for SkMel28, 10 μM idebenone for A375 and 50 nM idebenone for Mel Juso. For Western blot analysis of idebenone rescue adhered 2×10^5^ cells on 6 cm dishes in standard media were treated with 100 nM for SkMel28, 200 nM for A375, 200 nM for Mel Juso of idebenone. Cells were harvested 3 days post-transduction. For FACS (Bodipy C11) assays 2×10^5^ cells were treated in 6 cm dishes with 100 nM idebenone for SkMel28, 200 nM idebenone for A375 and 200 nM idebenone for Mel Juso. Cells were harvested 4 days post-transduction. For all treatments idebenone was added to cells at the same time with lentiviral transduction. Media supplemented with idebenone was replaced every 2 days.

### 2.5 Quantitative real-time PCR

Total RNA was isolated with TRIzol (Invitrogen). cDNA was synthesized by the High-Capacity cDNA Reverse Transcription Kit (Applied Biosystems). Real-Time Quantitative Reverse Transcription PCR (qRT-PCR) was performed using the double-stranded DNA dye SYBR Green (Roche, Switzerland) on CFX Real-Time PCR Detection System (Bio-Rad). Ct values were normalized to β-actin. Fold change expression was calculated using the ΔΔCt method. Sequences for all primers were as follows: forward, 5'-CACTTGGCTCTTATCTACTTTGGA-3', and reverse, 5'-GTCTCCCAGAGCCACGTACTTG-3' for UBIAD1; forward, 5'-CAGTATCCTGCCGAGTCTGT-3', and reverse, 5'-TGAACACTCGCTCAAACCAG-3' for NQO1; forward 5’- GGGGCTAGTAGTGGGGATAG-3’ and reverse 5’-TCCTCATAGGCCTGGATAGC-3’ for FSP1; forward 5’- ACTGAGCCTAGTGGGTGTGA-3’ and reverse 5’- GGATGTCTAGAGTGTAAATCTGGTG-3’ for COQ2; forward, 5'-GATGGAGTTGAAGGTAGTTTCGT-3', and reverse, 5'-GCGGGAAATCGTGCGTAGCATT-3' for β-actin as internal control.

### 2.6 Western blotting

For immunoblotting, cells were washed by 1× PBS and scraped on ice in RIPA buffer (Life Technologies) containing protease and phosphatase inhibitors (Complete Mini, Roche). Sonicated cell lysates were then centrifuged at 20,000 g at 4°C for 15 min. Protein concentration was determined using the mini BCA assay (Pierce Biotechnology) before being loaded to SDS-PAGE gel. Protein lysates were separated on 4–12% gradient SDS-PAGE (Thermo Fisher) gels and transferred to a 0.22 μM nitrocellulose membrane (Sigma). Membranes were blocked with 5% milk in 1× TBST solution for 1 h. Primary and secondary antibodies were diluted in 1% BSA. Incubation with primary antibodies was performed by shaking overnight at 4°C. Incubation with secondary antibodies (horseradish peroxidase (HRP)-conjugated anti-rabbit immunoglobulin G (IgG) (#A6154, Sigma, 1:10,000) and HRP-conjugated anti-mouse (IgG) (#A4416, Sigma, 1:10,000)) was performed by shaking at RT for 1 h. Following the incubation with both primary and secondary antibodies, all membranes were washed three times 5 min in 1× TBST. The bands were visualized and acquired using ChemiDoc™ Imaging System (Bio-Rad). The band intensities of proteins were quantified using Image Lab software (Bio-Rad Laboratories). Following antibodies and dilutions were used for immunoblotting: β-actin (#691331, MP Biomedicals, 1:4000), UBIAD1 (TERE-1 H-8) (#sc-377,013, Santa Cruz, 1:100), UBIAD1 (#HPA044862, Atlas Antibody, 1:500), NQO1 (A180) (#sc-32793, Santa Cruz Biotechnology [SCB], 1:1000), FSP1 (#sc-377,120, SCB, 1:1000), RRM2 (#65939, CST, 1:1000), cyclin A (#C4710, Sigma, 1:1000), pan-AKT (#4691, CST, 1:1000). p-AKT(Ser473) (#9271, CST, 1:1000), p-AMPKα1/2 (Thr172) (#sc-33524, SCB, 1:1000), t-AMPKα1/2 (CST, #5832), p-p65 (CST, #30315, 1:1000), p65 (CST, #3034, 1:1000), p-ERK1/2 (Thr202/Tyr204) (#9101, CST, 1:1000), t-ERK1/2 (#4695, CST, 1:1000), PMCA (#MA3-914, Thermo Fisher, 1:1000), Tom20 (#sc-11415, SCB, 1:1000).

### 2.7 Determination of cell death using AnnexinV/PI staining

2×10^5^ of cells were used for FACS analysis. Non-adherent cells were harvested by centrifugation at 800 g, 5 min. Adherent cells were trypsinized (Trypsin #ECB3052 EuroClone). Cells were combined and washed 3 times in ice-cold 1× PBS by subsequent centrifugation and resuspension of pellets (800 g, 5 min, at 4°C). Cells were stained according to the manufacturer's protocol (#4252, Santa Cruz). Staurosporine treatment (1 μM, 16 h) was used as positive control of apoptotic cell death. CH (component F in #C10446, Thermo Fisher) cell treatment (100 μM for 0.5 h) was used as control for cell death, induced by lipid peroxidation. 2×10^5^ cells were resuspended in 200 μL of AnnexinV-binding solution to obtain single-cell suspension. Samples were analyzed on a BD FACSCanto™ II Cell Analyzer (BD Biosciences) using the AlexaFluor 488 filter for the FITC labeled AnnexinV antibody and the PE-Cy5 filter for PI staining. Non-treated cells only with AnnexinV staining were used as blank. A minimum of 10,000 events were obtained per sample.

### 2.8 Co-localization studies of UBIAD1

1×10^5^ of cells were seeded on glass coverslips placed in a 24-well plate in DMEM complete medium and let adhere overnight. Cells were washed in 1× PBS 3 times, fixed by 4% paraformaldehyde for 10 min at RT, permeabilized with 0.2% Triton X-100 in 1× PBS for 10 min and blocked with solution containing 3% goat serum, 3% Albumin bovine serum (BSA) in 1× PBS for 1 h at RT. Incubation with primary antibodies diluted in 3% BSA in 1× PBS was performed for 1 h at RT. The following primary antibodies were used to detect UBIAD1: UBIAD1 (#HPA044862, Atlas Antibodies, 1:100) and TERE-1 H-8 (#sc-377,013, Santa Cruz, 1:100), to detect Golgi: GM-130 (#610822, BD Transduction Laboratories, 1:150) and to detect ER: calreticulin (#2907, Abcam, 1:100). Incubation with the corresponding Alexa Fluor conjugated secondary antibodies (2 μg/ml, Invitrogen) in 0.2% BSA in 1× PBS and DAPI for DNA staining (300 nM) was done for 1 h in the darkness. Cells were washed after incubation with both primary and secondary antibodies 3 times with 0.1% Triton X-100 in 1× PBS. Coverslips were mounted with Mowiol 4–88 (Sigma) and fixed on microscope slides (Thermo Fisher) with nail polish. Cells were imaged using 40× magnification Leica SP8 DLS microscopy.

### 2.9 Click-iT lipid peroxidation assay

Briefly, 1×10^5^ cells were seeded in standard medium on glass coverslips in 24-well plates and let adhere overnight. Lentiviral-mediated UBIAD1^KD^ was performed on attached cells. Lipid peroxidation assay was performed according to the manufacturer’s protocol of Click-iT staining (Thermo Fisher). DAPI staining for DNA visualization (300 nM) was done for 1 min in the darkness. Fluorescence was observed by Leica SP8 DLS microscopy using 40*x* magnification. Images were analyzed with Image J (National Institutes of Health).

### 2.10 Bodipy C11 lipid peroxidation assay

Lipid peroxidation was evaluated by flow cytometry using Bodipy C11 581/591 (Thermo Fisher). 1×10^5^ cells for ctrl and 2×10^5^ cells for UBIAD1^KD^ and NQO1^KD^ were seeded in standard medium in a 6 cm dish and let adhere for 6 h. UBIAD1 or NQO1 knockdown was induced by lentiviral transduction as previously described. After 4 days of infection cells were incubated for 15 min at 37°C in the dark with 2.5 μM Bodipy C11 in 1× PBS supplemented with 5% FBS. As positive control, cells were treated with 100 μM of cumene hydroperoxide (CH) for 30 min. Floating cells were collected and combined with adherent cells detached by trypsinization (Trypsin #ECB3052 EuroClone). Cellular pellets were washed 3 times with 1× PBS at 1000 g for 5 min at 4°C. Eventually, 2×10^5^ cells were resuspended in 200 μl of 1× PBS + 5% FBS and pipetted up and down to obtain a single-cell suspension. Flow cytometry analysis was performed using BD FACSCanto™ II Cell Analyzer (BD Biosciences). Non-treated cells without Bodipy C11 staining were used as blank. The ratio between oxidized (FITC-A) and reduced (PE-A) Bodipy C11 was measured. A minimum of 20,000 events were obtained per sample.

### 2.11 ROS analyses by DHE and DCFH-DA assay

Intracellular ROS levels were measured by flow cytometry after cells were stained with dihydroethidium (DHE) (#D23107, Thermo Fisher) (for superoxide) and dichlorodihydrofluorescein diacetate (DCFH-DA) (#C6827, Thermo Fisher) (for H_2_O_2_).

1×10^5^ cells for ctrl and 2×10^5^ cells for UBIAD1^KD^ and NQO1^KD^ were seeded in standard media in 6 cm dishes and let adhere for 6 h. UBIAD1 or NQO1 knockdown was induced by lentiviral transduction as previously described. After 4 days of silencing cells were stained with DHE or DCFH-DA, according to manufacturer’s recommendations: floating and attached cells were harvested by trypsin (Trypsin #ECB3052 EuroClone), rinsed with 1× cold-PBS, and then probed with DHE (10 μM) and DCFH-DA (300 nM) in 1× PBS+5% FBS for 10 min at 37°C in the dark. Together with DHE or DCFH-DA staining, cells were incubated with Fixable Viability Stain 780 (#565388, BD) 0.5 μl/1×10^6^ cells for discrimination of viable and non-viable cells. Treatment with menadione (50 μM) and H_2_O_2_ (1 mM) for 90 min was used as positive control for DHE and DCFH-DA respectively. 2×10^5^ cells were resuspended in 500 μl of 1× PBS + 5% FBS and pipetted up and down to obtain a single-cell suspension. Flow cytometry analysis was performed using BD FACSCanto™ II Cell Analyzer (BD Biosciences). No stained cells were used as blank. Singlet stained DHE (PE-A), DCFH-DA (FITC-A) and FVS 780 (APC-Cy7-A) were used for fluorescence compensation. A minimum of 20,000 events were recorded per sample.

### 2.12 Apoptotic DNA fragmentation TUNEL assay

Apoptotic cells were detected by APO-BrdU™ TUNEL Assay Kit, with Alexa Fluor™ 488 Anti-BrdU (#A23210, ThermoFisher). 1×10^5^ cells for ctrl and 2×10^5^ cells for UBIAD1^KD^ were seeded in standard medium in 6 cm dishes and let adhere for 6 h. UBIAD1 knockdown was induced by lentiviral transduction as previously described. After 4 days of silencing cells were stained according to manufacturer’s recommendations: floating cells were collected and combined with adherent cells detached by trypsinization. Cells were washed once in 1× PBS and fixed for 15 min on ice with 1% (w/v) PFA in 1× PBS. Then, cells were washed twice with 1× PBS, resuspended in ice-cold 70% (v/v) ethanol and incubated at −20 °C for 2 h. After ethanol removal, samples were washed twice with wash buffer (blue cap) and resuspended in DNA-labeling solution. To obtain the best staining, ¼ of the suggested reagent concentrations were used and incubation time was decreased to 15 min at 37°C. At the end of incubation time, cells were washed twice with rinse buffer (red cap) and incubated 30 min at room temperature protected from light with Alexa Fluor 488 dye-labeled anti-BrdU antibody (to obtain the best staining, ¼ of the suggested antibody concentration was used). Then, cells were washed once in 1× PBS and resuspended in 500 μl of 1× PBS. All centrifugations were performed for 5 min at 4°C at 1000 g. Samples were analyzed within 3 h of completing the staining procedure using BD FACSCanto™ II Cell Analyzer (BD Biosciences) using FITC-A filter. A minimum of 50,000 events were collected per sample. As positive control, ctrl cells were treated with staurosporine (1 μM) for 16 h before proceeding with the staining procedure. Not stained control cells were used as blank.

### 2.13 Subcellular fractionation for plasma membrane CoQ10 measurements

2×10^5^ cells for ctrl and 1×10^6^ cells for UBIAD1^KD low^ were seeded in 10 cm dishes and let adhere for 6 h. UBIAD1 knockdown was induced by lentiviral transduction as previously described. After 5 days, cells were collected and washed 3 times with ice-cold 1× PBS by centrifugation at 4°C at 600 g for 10 min. All the following steps were performed on ice. Pellets were resuspended using 500 μl of ice-cold 1× PBS containing 0.1 M Tris-HCl pH 7.4, 0.1 M EGTA, 1 M sucrose and protease/phosphatase inhibitors (Complete Mini, Roche) and incubated 30 min on ice. Ten μl were saved, resuspended in RIPA buffer (#89900, Thermo Fisher) as Whole Cell fraction (W). Cells were homogenized using a pre-chilled potter (#RTCXH1.1, ROTH) till 80% of cells were lysed (approximately 40 strokes for A375 and 45 strokes for SkMel28). Percentage of cell lysis was checked using Trypan Blue staining. Cell lysates were centrifuged twice at 600 g for 5 min at 4°C to remove unbroken cells and nuclei (pellets). Supernatants were centrifuged at 8,000 g for 20 min at 4°C to obtain a pellet formed by crude mitochondria and a supernatant enriched in cytosolic organelles and plasma membrane. The pellet was then resuspended in 2 mM MgCl_2_-containing 1× PBS and centrifuged at 3,000 g for 15 min at 4°C to pellet more pure mitochondria. Pellets were resuspended in RIPA buffer to form the Mitochondria fraction (M). The supernatants containing the cytosolic organelles and plasma membranes were centrifuged at 21,000 g for 90 min at 4°C. Pellets containing plasma membrane and plasma membrane associates were resuspended in ice-cold PBS containing 0.1 M Na_2_CO_3_ and 1 mM EDTA (pH 11.3) for 30 min on ice. Final centrifugation was performed at 21,000 g for 99 min at 4°C. Plasma membrane pellets were resuspended in 30 μl of PBS forming the Plasma membrane fraction (PL). One third of the volume was saved and resuspended with RIPA buffer for Western blot analysis, while the remaining 2/3 were frozen in liquid nitrogen and stored at −80°C for mass spectrometry analysis.

### 2.14 UHPLC-MC/MS analyses of CoQ10 level

Cells were seeded in 10 cm plates in DMEM supplemented medium and let to reach 80% of confluence. Cells were washed with ice-cold 1× PBS 3 times and scraped in 200 μl of 1× PBS on ice. Part of the suspension (20 μl) was used to measure protein concentration by BSA method and WB analysis to check UBIAD1 knockdown. The remaining volume (180 μl) was immediately frozen in dry ice and stored at −80°C. For the extraction of CoQ10 30 μg of protein were transferred to 300 μl of ice-cold extraction solution (ethanol/hexane 1:2) with 0.2 μM of CoQ9 used as internal standard followed by vortexing. Cells were then centrifuged at 13,200 g for 5 min at 4°C. The upper phase was transferred to a glass mass spectrometry vial. The extraction procedure was performed 2 times. The hexane was dried under a stream of N_2_ and dried samples were resuspended in 100 μl of methanol. To obtain the calibration curves, working calibration solutions of CoQ10 within range 2–1000 nM were diluted serially from a stock solution of CoQ10 in methanol. Internal standard of CoQ9 was spiked in each calibration standard at a concentration of 0.2 μM. The instrument was calibrated before the analysis using a commercial calibration solution to maintain mass accuracy below 5 ppm. CoQs were determined as [M+H]+ and [M + NH4]+ adducts ions. Quantification of CoQ10 concentration was done relative to the internal standard by an external calibration curve. Results are presented as ng of CoQ10 per mg of proteins. The UHPLC/MS analysis was done on a Hybrid quadrupole-Orbitrap system (Q Exactive, Thermo Scientific) coupled to an UHPLC system (Ultimate 3000, Thermo Dionex) via a heated electrospray ionization source. Four μl of each sample was injected in the Accucore C18 100 × 2.1 (2.6 μm particle size) column. (Thermo Fischer Scientific) The UHPLC--/MS was operated at a flow rate of 250 μl/min with a gradient elution of 10 mM of ammonium formate in water (phase A) and 10 mM of ammonium formate in methanol/2-propanol 80:20 (phase B). Data were acquired in full MS/ddMS2mode with positive electrospray ionization mode. Similar procedure was used to analyze CoQ10 level in plasma membrane fraction. Ceramide 16:0 (Cer16:0) was used as internal control to normalize the CoQ10 amount among samples.

### 2.15 Lipid class quantification by Gas-Chromatography(GC) and GC-MS analyses

The following internal standards were used: trinonanoylglycerol and tripentadecanoylglycerol for triacylglycerols (TG), 5-α-cholestane for cholesteryl esters (CE), (3α, 5β)- cholestan-3-ol (epicoprostanol) for free cholesterol (CO). Lipids were extracted from 100 μl of sample using a chloroform-methanol solution in accordance with the Folch method (Folch J 1957). Extracted lipids were separated to classes by thin layer chromatography as previously reported [23]. The TG fraction was hydrolyzed with 2 ml of HCl-methanol (5%) (Merck). The resulting free fatty acids were *trans*-esterified and extracted with hexane. Hexane containing fatty acid methyl esters (FAME) was directly used for GC analysis on Agilent 5890 gas chromatograph (GC) equipped with a flame-ionization detector. FAMEs derived from TG were resolved with an Omegawax column (30 m × 0.25 mm internal diameter x 0.25 μm film thickness; Supelco) with injection of 1 μl running in on-column mode. The program for oven temperature was set up next: 60 °C for 3 min, increased 20°C/min to 205°C, then remained constant for 15 min. Temperature then increased 0.4°C/min up to 213°C, which was maintained for 10 min and finally increased to 240°C at 5.0 °C/min and held for 8 min. Peaks were determined in relation to a reference standard mixture (GLC 461, Nuchek Prep).

CO and CE were eluted from the silica with 5 ml of mixture of chloroform-methanol 2:1. The solvent was collected and evaporated to dryness under a stream of N_2_. CE have been hydrolyzed by saponification and obtained free CO were extracted with hexane. In the end, both dried residues containing CO were derivatized in 50 μl of N,O-Bis(trimethylsilyl)trifluoroacetamide (BSTFA) (Merck) for 30 min at RT. 1 μl of the resulting mixture was injected for GC-MS analysis. Measurements of sterols were recorded by gas chromatography-mass spectrometry (GC-MS) instrument Agilent 6890 GC coupled with Agilent 5973 inert MS on the single ion monitoring (SIM) mode. Splitless mode was set up for the samples injection at 270°C. Separation was done using an HP-5ms column (30mx0.25 mm internal diameter × 0.25 μm film thickness; Agilent Technologies, Folsom, CA, USA). The oven temperature was set up initially at 200°C for 1 min, raised to 275°C at 10°C/min, then to 277°C at 0.1°C/min and finally increased to 290°C at 10 °C/min and was held for 3 min. Determination of peak was done by comparing the retention time and matching the height ratios of the characteristic ions. The internal standard method was used for quantification. 25 mm.

### 2.16 UBIAD1 expression in human cutaneous melanoma patient cohort

Publicly available RNA expression levels and associated clinico-pathological and survival data of the skin cutaneous melanoma patients enrolled in the TCGA-SKCM cohort [24] were retrieved from cBioPortal [25,26]. Out of 470 patients, 10 were excluded from the analysis because of missing “Overall Survival” information or RNA expression data. Three patients had more than one biological sample available, the RNA expression profile from the sample with the lowest “SAMPLE_ID” was retained since it matched the “primary tumor” sample when both primary and metastatic samples were sequenced. Patients were then stratified according to UBIAD1 RNA expression levels in two groups: High, UBIAD1 RNA expression level above or equal to the median; Low, UBIAD1 RNA expression level below the median. The hazard ratio was calculated with a 95% confidence interval (95% CI) using Cox proportional Hazard regression both in univariable and in multivariable models to adjust for all the standard clinicopathological parameters by the “coxph” function of the “survival” package [27] in R [28]. The proportionality assumption of the hazards over time was tested with the Schoenfeld test as implemented in the “cox.zph” function of the “survival” package; none of the variables considered in the model violated the proportionality assumption. The forest plot was generated with the “forestmodel” package [29].

### 2.17 Statistical analyses

GraphPad Prism 8 was used for statistical analysis. For 2 experimental comparisons, 2-tailed unpaired Student’s t-test was used. For multiple comparisons, 1- and 2-way ANOVA and 1-sample *t*-test were applied. Specified number of biological replicates are defined in the legends. A p-value of less than 0.05 was considered significant. Statistical significance is reported as exact p-value or ns, when not significant.

## 3 Results

### 3.1 High UBIAD1 expression is associated with poor survival (OS) of melanoma patients and with melanoma cell lines

**Fig. 1:**
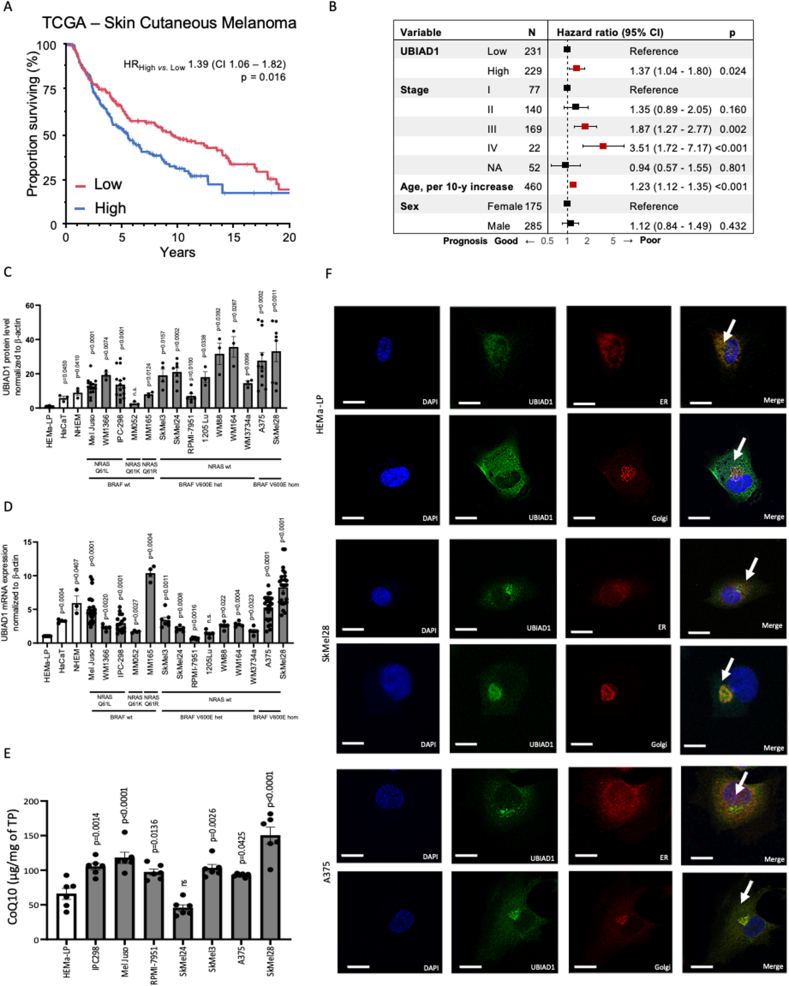
High UBIAD1 expression is associated with poor survival of melanoma patients and with melanoma cell lines. (A) Kaplan-Meier survival plot (truncated at 20 years) showing the proportion of surviving patients (OS) stratified according to the UBIAD1 mRNA expression levels (High vs. Low) in the TCGA-SKCM cohort. HR, univariable Hazard Ratio; CI, 95% Confidence Interval; p, p-value. (B) Forest plot showing multivariable hazard ratios, 95% confidence intervals (CI), and p values for the association between the indicated factors and good (HR < 1) or bad (HR > 1) prognosis in patients stratified by their UBIAD1 (High vs. Low) mRNA expression level. NA, Not Available. (C) Comparison of UBIAD1 protein level in different human melanocytes (NHEM, HEMa-LP), human epidermal keratinocyte (HaCaT) and melanoma cell lines. UBIAD1 level was normalized to β-actin and reported as relative to HEMa-LP (set at 1). One-sample *t*-test (hypothetical mean = 1) was used to quantify statistical significance. Error bars represent SEM, n ≥ 3. NRAS and BRAF mutation status was reported under each melanoma cell line: wt, wild type; het, heterozygous and hom, homozygous. (D) qRT-PCR quantifications of UBIAD1 mRNA levels in a panel of cell lines. UBIAD1 mRNA level was normalized to β-actin and reported as relative to HEMa-LP (set at 1). One-sample *t*-test (hypothetical mean = 1) was used to quantify statistical significance. Error bars represent SEM, n ≥ 3. NRAS and BRAF mutation status was reported under each melanoma line: wt, wild type; het, heterozygous and hom, homozygous. (E) HPLC-MS analyses of CoQ10 in different melanoma cell lines, normalized to total protein (TP) concentration. 1-w ay ANOVA with Dunnett‘s multiple comparisons test (HEMa-LP as a control) was used to quantify statistical significance. Error bars represent SEM, n = 6. (F) Subcellular co-localization of UBIAD1. Confocal images showed prevalent UBIAD1 co-localization with ER marker calreticulin in HEMa-LP melanocytes and prevalent UBIAD1 co-localization with Golgi marker GM130 in melanoma cell lines SkMel28 and A375. Image scale bars = 15 μm.

The role of the UBIAD1 metabolic enzyme in cancer progression has been poorly investigated. To define the role of UBIAD1 in melanoma progression and in the biology of real-life melanoma tumors, we evaluated the prognostic potential of its transcriptional levels in the TCGA Skin Cutaneous Melanoma (SKCM) cohort, which represents until now the largest collection of clinico-pathological information of SKCM patients with associated publicly available transcriptomics data (Fig. 1A). We observed a statistically different probability of overall survival (OS) in patients dichotomized at the median expression level of *UBIAD1*, with the group with the higher expression demonstrating a worse prognosis as compared to the group with lower expression (HR = 1.39; 95% CI = 1.06–1.82; p-value = 0.016). The poor prognostic behavior of the UBIAD1^high^ group persisted even in multivariable analysis (HR = 1.37; 95% CI = 1.04–1.8; p-value = 0.024) after correcting for standard prognostic factors such as tumor stage, age, and sex of the patient (Fig. 1B), thus defining the expression level of UBIAD1 as an independent prognostic factor.

Next, we investigated UBIAD1 expression across multiple human melanoma cell lines both at protein and mRNA levels. As control we analyzed normal human melanocytes (HEMa-LP and NHEM) and immortalized epidermal cells (HaCaT). We observed a significant expression of UBIAD1 protein in most of the melanoma cells tested, with BRAF-mutated cell lines (except for RPMI-7951) showing the highest amount of UBIAD1 (Fig. 1C and Fig. S1A). UBIAD1 mRNA levels fluctuate among melanoma cells with the MM165, A375 and SkMel28 showing the highest levels (Fig. 1D). Intriguingly, the highest expression levels of UBIAD1 both at mRNA and protein levels were detected in SkMel28 and A375.

UBIAD1 enzyme is responsible for the biosynthesis of the non-mitochondrial pool of CoQ10 and it plays an important role as an antioxidant enzyme [22]. Using high resolution mass-spectrometry we measured the levels of CoQ10 in melanocytes and melanoma cells (Fig. 1E). Compared to melanocytes, CoQ10 level is elevated in all melanoma cell lines except for SkMel24. Since CoQ10 could be also synthesized by COQ2, the mitochondrial homologue of UBIAD1, we analyzed mRNA expression of COQ2 in these cells (Fig. S1B). Overall, we observed that COQ2 mRNA level is similarly expressed by melanocytes and other melanoma cells, except for IPC-298, SkMel3 and A375 where COQ2 mRNA is significantly upregulated. Looking at the discrepancy between COQ2 and UBIAD1 mRNA levels in different cell lines, we conclude that there is no compensatory nor synergistic transcriptional regulation of CoQ10 synthesizing enzymes.

UBIAD1 can be localized both in the ER and the Golgi apparatus as previously demonstrated [22,30]. Such different localization has been associated with different functions of UBIAD1: in the ER it binds and stabilizes 3-hydroxy-3-methylglutaryl coenzyme A reductase (HMGCR), a key rate-limiting enzyme involved in cholesterol synthesis [30], while in Golgi it participates in CoQ10 synthesis [22]. We thus examined the subcellular localization of UBIAD1 in melanoma cell lines SkMel28 and A375 and in normal melanocytes HEMa-LP (Fig. 1F). Immunofluorescence data showed that UBIAD1 is mainly co-localized to the Golgi in melanoma cell lines compared to healthy melanocytes where it is mainly present in ER.

Overall, we conclude that UBIAD1 and CoQ10 are significantly expressed during melanoma progression as well as in melanoma cells carrying both BRAF and NRAS mutations. These results open the question whether UBIAD1 and CoQ10 might represent an important part of the redox systems which characterize melanoma progression and melanoma cells [5].

### 3.2 UBIAD1 loss impairs melanoma cell proliferation and survival by reducing plasma membrane CoQ10

**Fig. 2:**
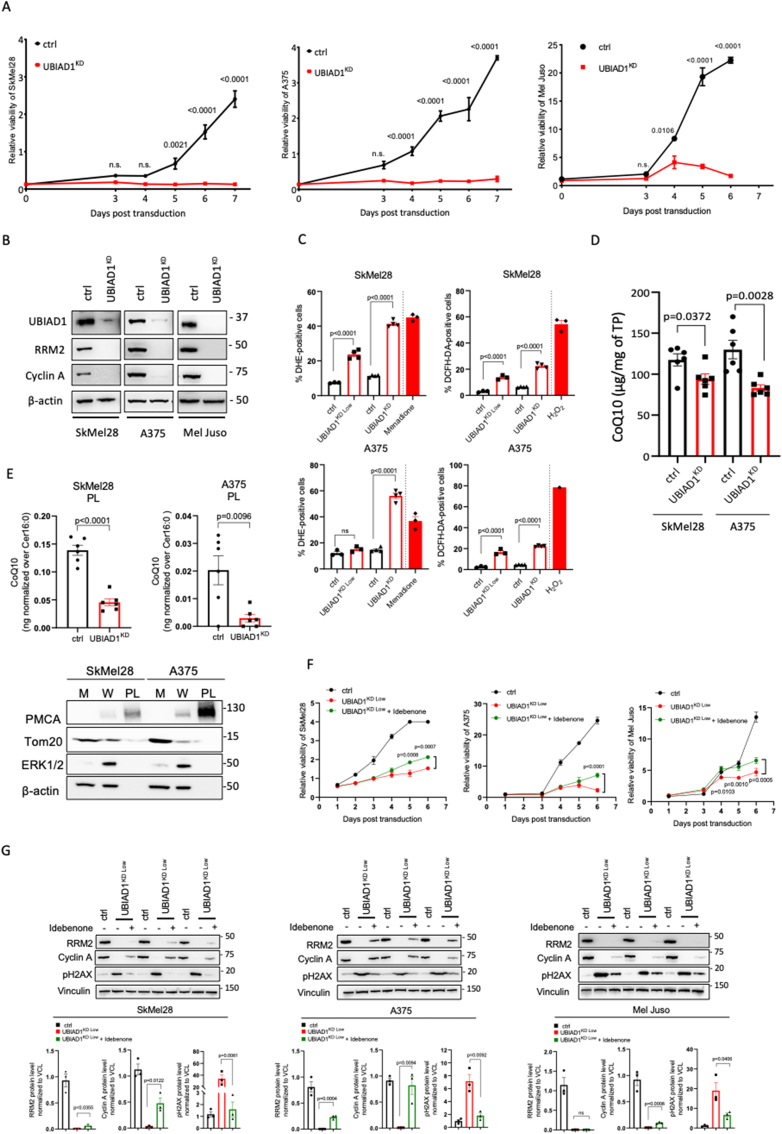
UBIAD1 loss leads to ROS increase, impaired cell proliferation and decreased survival in melanoma cells. (A) Growth curves of melanoma cell lines SkMel28, A375 and Mel Juso upon UBIAD1^KD^ compared to control conditions (ctrl). 2-way ANOVA with Sidak's multiple comparisons test was used to quantify statistical significance. Error bars represent SEM, n = 3. (B) Western blot analysis of cyclin A and RRM2 proteins upon UBIAD1^KD^ in SkMel28, A375 and Mel Juso melanoma cell lines. β-actin was used as loading control. (C) FACS assay of DHE (left) and DCFH-DA (right) staining in SkMel28 and A375 upon UBIAD1^KD Low^ or UBIAD1^KD^. Data are reported as percentage of positive cells over singlets. Incubation with 50 μM of menadione and 1 mM of H_2_O_2_ for 90 min were used as positive controls for DHE and DCFH-DA, respectively. One-way ANOVA with Sidak’s multiple comparisons test was used to quantify statistical significance. Error bars represent SEM, n ≥ 3. (D) HPLC-MS analyses of total CoQ10 level upon UBIAD1^KD^ in SkMel28 and A375 lines, in comparison control conditions (ctrl), normalized to total protein (TP) concentration. Unpaired Student two-tailed *t*-test was used to quantify statistical significance between control and UBIAD1^KD^ samples. Error bars represent SEM, n = 6. (E) HPLC-MS analyses of CoQ10 in plasma membrane fraction (PL) upon UBIAD1^KD^ in SkMel28 and A375 lines normalized to membrane ceramide abundance (Cer16:0). Unpaired Student two-tailed *t*-test was used to quantify statistical significance between control conditions (ctrl) and UBIAD1^KD^ samples. Error bars represent SEM, n = 6. Below, Western blot analysis of subcellular fractionation in SkMel28 and A375 lines. M, Mitochondria-enriched fraction; W, Whole cell; PL, Plasma membrane-enriched fraction. PMCA was used as marker of plasma membrane, Tom20 as mitochondrial marker, ERK1/2 and β-actin as cytosolic markers. (F) Growth curves of melanoma cell lines SkMel28, A375 and Mel Juso upon UBIAD1^KD Low^ in presence or absence of idebenone treatment (1 μM idebenone for SkMel28, 10 μM idebenone for A375 and 50 nM idebenone for Mel Juso). 2-way ANOVA with Sidak's multiple comparisons test was used to quantify statistical significance between UBIAD1^KD Low^ and UBIAD1^KD Low^ + idebenone. Error bars represent SEM, n = 3. (G) Western blot analysis of cell proliferation markers (RRM2 and cyclin A) and DNA-damage marker (pH2AX) in melanoma cell lines SkMel28, A375 and Mel Juso upon UBIAD1^KD Low^ in presence or absence of idebenone (100 nM of idebenone for SkMel28, 200 nM of idebenone for A375 and 200 nM idebenone for Mel Juso). Three biological replicates are shown. Unpaired Student two-tailed *t*-test was used to quantify statistical significance between UBIAD1^KD Low^ and UBIAD1^KD Low^ + idebenone samples. Error bars represent SEM, n = 3.

To understand the function of UBIAD1 in melanoma, we focused our studies on SkMel28 and A375 cell lines, carrying the BRAF^V600E^-mutation, and the Mel Juso cell line, carrying the NRAS^Q61L^-mutation. We then knocked-down UBIAD1 (UBIAD1^KD^) in these cell lines and performed a growth curve assay to determine whether UBIAD1 loss could affect melanoma proliferation. UBIAD1^KD^ resulted in a significant reduction of cell viability in these cell lines with strongest effects on SkMel28 and A375 cell lines (Fig. 2A). To confirm these data, we analyzed the level of two markers of cell proliferation: cyclin A and ribonucleotide reductase subunit M2 (RRM2) a cell-cycle-regulated enzyme, that catalyzes the rate-limiting step in the *de novo* synthesis of DNA precursors [31] (Fig. 2B). Both cyclin A and RRM2 levels dropped in UBIAD1^KD^ conditions, confirming proliferation defects in UBIAD1^KD^ cells.

To further understand the effect of UBIAD1^KD^ in melanoma cells we examined a series of signaling pathways which play key roles in protein synthesis, survival, proliferation and metabolism (Figs. S2A–F) [[32], [33], [34], [35]]. Compared to control cells, UBIAD1^KD^ affects phosphorylation of AKT(Ser473) protein, S6 signaling and AMPK in SkMel28, while UBIAD1^KD^ affect significantly only AKT and mTOR/s6 signaling in A375. Compared to control cells, UBIAD1^KD^ decreased the protein level of AKT and ERK1/2 in SkMel28 and Mel Juso, but not in A375. Hence, we observed different responses in all three cell lines. This experimental evidence raises the question of the involvement of different signaling pathways in each cell line and remains for now inconclusive.

UBIAD1 exerts an antioxidant function in cardiovascular tissues [22]. Thus, to better characterize the mechanism involved in UBIAD1-mediated survival of melanoma cells, we analyzed ROS levels in UBIAD1^KD^ cells (Fig. 2C). Our results showed that UBIAD1 loss increased ROS levels evaluated as DHE and DCFH-DA staining in both SkMel28 and A375 cells in a dose-dependent manner. Interestingly we noticed that non-viable UBIAD1^KD low^ and UBIAD1^KD^ were mainly positive to DHE staining that detects cytosolic superoxide, ONOO and •OH (Fig. S2G), while viable UBIAD1^KD low^ and UBIAD1^KD^ cells were mainly positive to DCFH-DA that detects hydrogen peroxide, hydroxyl radical, carbonate radical, and nitrogen dioxide (Fig. S2H) [36]. This suggests a temporal cascade in the generation of different reactive redox species that upon UBIAD1 knockdown leads to cell death.

Our group previously demonstrated that, being co-localized in the Golgi compartment, UBIAD1 is responsible for the biosynthesis of non-mitochondrial/plasma membrane CoQ10 [22]. Since our co-localization studies showed that UBIAD1 is mostly present in Golgi apparatus in BRAF-mutated melanoma cells, we examined CoQ10 levels after UBIAD1^KD^ in SkMel28 and A375 cells and we found them to be significantly reduced, as expected (Fig. 2D). To support the functional role of the non-mitochondrial fraction of CoQ10 after UBIAD1^KD^, we isolated plasma membrane fractions (PL) from melanoma cells and measured CoQ10 levels (Fig. 2E). Here we detected a significant and important drop of CoQ10 in UBIAD1^KD^ cells compared to controls. Since UBIAD1 has been also associated with the regulation of cholesterol synthesis due to its putative ability to bind and stabilize the cholesterol biosynthetic enzyme HMGCR [37,38], we also measured free cholesterol (CO), cholesterol esters (CE) and triacylglycerols (TGs) (Fig. S2I). Surprisingly, UBIAD1^KD^ did not alter TGs, CE nor CO levels in these melanoma cells.

To confirm that the loss of UBIAD1-dependent CoQ10 synthesis is responsible for cell viability defects, we performed a rescue-viability assay by treating UBIAD1^KD^ cells with idebenone, a synthetic analog of CoQ10 with increased solubility, to emulate the same physiological mechanism of reduced CoQ10 [13]. Treatment with idebenone resulted in a significant rescue of the viability of SkMel28, A375 and Mel Juso after a mild KD of UBIAD1 (UBIAD1^KD Low^) (Fig. 2F). To further support that the CoQ10 analog, idebenone, is able to rescue or preserve cell viability and thus proliferation, we analyzed the level of two markers of cell proliferation, cyclin A and RRM2, as well as a marker of DNA damage (e.g. double-strand DNA breaks) as pH2AX (Fig. 2G). We found that idebenone treatment in UBIAD1^KD^ cells can significantly restore expression of these markers in all melanoma cell lines tested (except for RRM2 in Mel Juso), supporting the functional role of CoQ10 in UBIAD1-dependent melanoma survival.

Altogether, these data demonstrate that UBIAD1 is required to sustain survival and that it exerts this effect through the synthesis of CoQ10 in melanoma cells.

### 3.3 UBIAD1-mediated CoQ10 synthesis protects melanoma cells from lipid peroxidation

**Fig. 3:**
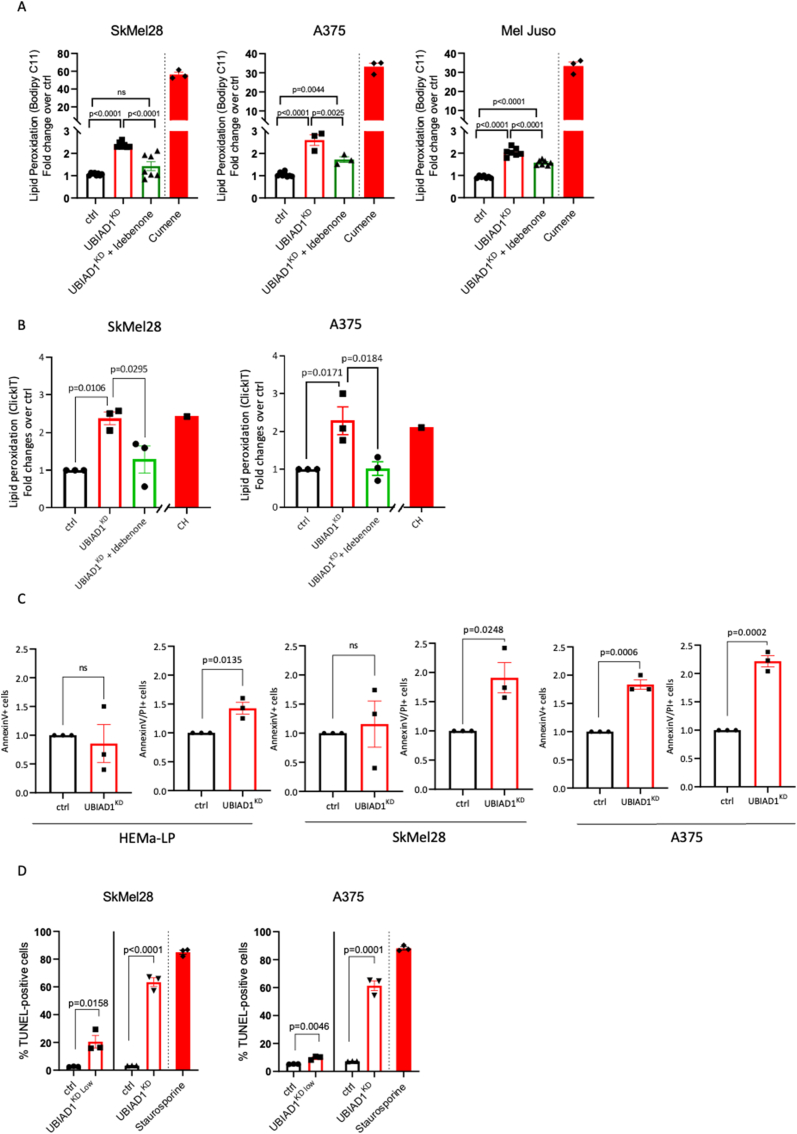
**UBIAD1 and plasma membrane CoQ10 protect melanoma cells from lipid peroxidation and apoptotic cell death.** (A) Lipid peroxidation assay using Bodipy C11 581/591 FACS upon UBIAD1^KD^ in presence or absence of idebenone treatment (100 nM of idebenone for SkMel28, 200 nM of idebenone for A375 and 200 nM of idebenone for Mel Juso). Ratio between oxidized and reduced Bodipy C11 was measured and reported as fold change over control conditions (ctrl). For positive control, cells were treated for 30 min with 100 μM of cumene hydroxyperoxide (CH). One-way ANOVA with Tukey's multiple comparisons test was used to quantify statistical significance. Error bars represent SEM, n ≥ 3. (B) Quantification of lipid peroxidation in melanoma cells, induced upon UBIAD1^KD^, assessed by Click-iT method. Statistical significance was quantified by 1-way ANOVA using Sidak's multiple comparisons test. Error bars represent SEM, n = 3. (C) Relative viability after UBIAD1^KD^ in melanocytes (HEMa-LP) and melanoma cells assessed by AnnexinV/PI flow cytometry after lentiviral transduction. Alive cells are defined as events negative for both PI and AnnexinV. Error bars represent SEM, n = 3. (D) Analysis of apoptosis by FACS TUNEL assay in melanoma lines SkMel28 and A375 upon UBIAD1^KD Low^ and UBIAD1^KD^. Data are shown as % of apoptotic cells (TUNEL-positive) over singlets. As positive control, cells were treated with 1 μM of staurosporine for 16 h. Unpaired Student two-tailed *t*-test was used to quantify statistical significance between ctrl and UBIAD1^KD Low^ or UBIAD1^KD^ samples. Error bars represent SEM, n = 3.

Reduced CoQ10 acts as a lipophilic radical-trapping antioxidant which detoxifies lipid peroxyl radicals [39]. To explore whether UBIAD1 deficiency induces lipid peroxidation in melanoma cells, we performed lipid peroxidation analyses using Bodipy C11 581/591 on SkMel28, A375 and Mel Juso cells after UBIAD1^KD^ (Fig. 3A). As positive control for lipid peroxidation we used cumene hydroperoxide (CH), since it can initiate and propagate lipid peroxidation [40]. UBIAD1^KD^ promoted significant lipid peroxidation in all melanoma cell lines. We also showed lipid peroxidation defects exerted by UBIAD1 loss in attached and living cells using Click-IT technology (Fig. 3B). Remarkably, UBIAD1 loss promotes lipid peroxidation in all tested melanoma cells lines. To demonstrate that the drop of CoQ10 that follows UBIAD1^KD^ is responsible for the induction of lipid peroxidation and thus of cell death, we performed rescue experiments by treating melanoma cells with idebenone (Fig. 3A–B). We observed that idebenone treatments were sufficient to rescue UBIAD1^KD^-mediated lipid peroxidation as evaluated by Bodipy C11 and Click-iT lipid peroxidation assays.

Recent data showed that CoQ10 depletion leads to ferroptotic cell death through the induction of lipid peroxidation [13]. We thus investigated whether UBIAD1^KD^ leads to ferroptosis. First, we performed AnnexinV/Propidium Iodide (PI) cell death staining. Analysis of AnnexinV/PI staining by flow cytometry allows to distinguish between “early apoptotic cells”, which are AnnexinV-positive and PI-negative (AnnexinV^+^/PI^−^) and “late apoptotic cells”, which are instead AnnexinV^+^/PI^+^. Compared to control cells, UBIAD1^KD^ led to a significant increase of AnnexinV^+^/PI^−^ signal in A375, but not in SkMel28 (Fig. 3C). On the contrary, both melanoma cell lines showed a highly significant increase of AnnexinV^+^/PI ^+^ staining after UBIAD1^KD^. We also observed that in healthy melanocytes UBIAD1^KD^ did not affect AnnexinV ^+^ cells, but only slightly affected AnnexinV^+^/PI ^+^ cells. These data indicate that UBIAD1^KD^ can induce cell death in all cell types tested but with a stronger effect in melanoma cells compared to normal melanocytes.

To unequivocally confirm that UBIAD1 loss promotes apoptotic cell death, UBIAD1^KD^ melanoma cells were tested by TUNEL assay, which measures the level of nucleosomal DNA-fragmentation, a strong hallmark of apoptosis (Fig. 3D). SkMel28 and A375 melanoma cells were TUNEL-positive after UBIAD1^KD^ with UBIAD1 loss leading to apoptotic cell death in a dose-dependent manner.

These results suggest that by lowering the amount of plasma membrane CoQ10, UBIAD1^KD^ promotes the initiation and propagation of lipid peroxidation in melanoma cells and their subsequent apoptotic (but not ferroptotic) cell death.

### 3.4 NQO1 suppresses lipid peroxidation by regenerating the antioxidant form of CoQ10

**Fig. 4:**
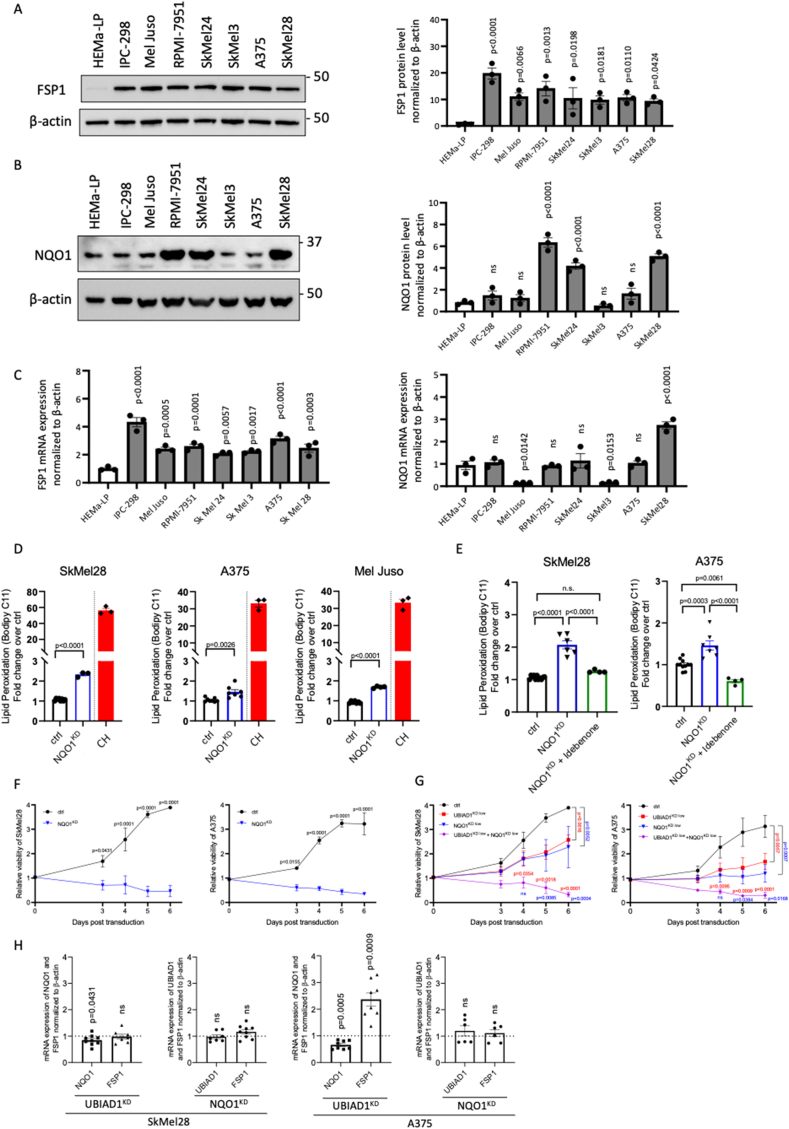
NQO1 is required to suppress lipid peroxidation by regeneration of the antioxidant form of CoQ10 in melanoma cells. (A–B) Western blot analyses and relative quantifications of FSP1 (A) and NQO1 (B) protein levels in a panel of melanoma cell lines, normalized to protein level in melanocytes (HEMa-LP). 1-way ANOVA with Dunnett’s multiple comparisons test (HEMa-LP as a control) was used to quantify statistical significance. Error bars represent SEM, n ≥ 4. (C) mRNA expression of FSP1 and NQO1 in a panel of melanoma cell lines. 1-way ANOVA with Dunnett‘s multiple comparisons test (HEMa-LP as a control) was used to quantify statistical significance. Error bars represent SEM, n = 3. (D) Lipid peroxidation assay using Bodipy C11 581/591 FACS upon NQO1^KD^ in melanoma cell lines SkMel28, A375 and Mel Juso. Ratio between oxidized and reduced Bodipy C11 was measured and reported as fold change over control conditions (ctrl). As positive control, cells were treated for 30 min with 100 μM of CH. Unpaired Student two-tailed *t*-test was used to quantify statistical significance. Error bars represent SEM, n ≥ 3. (E) Idebenone rescue of lipid peroxidation upon NQO1^KD^. Lipid peroxidation was assessed using Bodipy C11 FACS upon NQO1^KD^ in melanoma cell lines SkMel28 and A375 in presence or absence of idebenone (100 nM of idebenone for SkMel28, 200 nM of idebenone for A375). Ratio between oxidized and reduced Bodipy C11 was measured and reported as fold change over control conditions (ctrl). One-way ANOVA with Tukey's multiple comparisons test was used to quantify statistical significance. Error bars represent SEM, n ≥ 3. (F) NQO1 knockdown impairs viability of melanoma cells. Growth curves of melanoma cell lines SkMel28 and A375 upon NQO1^KD^ compared to control conditions (ctrl). 2-way ANOVA was used to quantify statistical significance. Error bars represent SEM, n = 3. (G) Co-silencing of NQO1 and UBIAD1 dramatically impairs viability of melanoma cells. Growth curves of melanoma cell lines SkMel28 and A375 upon mild NQO1^KD^ (NQO1^KD Low^), mild UBIAD1^KD^(UBIAD1^KD Low^), or simultaneous NQO1^KD Low^ + UBIAD1^KD Low^ compared to control conditions (ctrl). 2-way ANOVA was used to quantify statistical significance. Error bars represent SEM, n ≥ 3. (H) qRT-PCR quantifications of UBIAD1, NQO1 and FSP1 mRNA levels in UBIAD1^KD^ or NQO1^KD^ melanoma cells. One-sample *t*-test (hypothetical mean = 1) was used to quantify statistical significance. Error bars represent SEM, n ≥ 6.

Our data suggested a fundamental role of plasma membrane CoQ10 as an antioxidant preventing melanoma cell lines from lipid damage. To further investigate the mechanism of CoQ10-mediated lipid protection, we focused our attention on the role of NQO1 and FSP1, two plasma membrane NADH-dependent oxidoreductases. NQO1 possesses NAD(P)H:ubiquinone oxidoreductase activity and functions as a component of the plasma membrane redox system generating the reduced forms of CoQ10 (CoQ10H_2_ or ubiquinol) [41]. It has been shown that NQO1 plays a key role in melanomagenesis and is highly expressed in melanoma [42,43]. FSP1 has been recently identified as an NADH-dependent oxidoreductase localized to the plasma membrane where it mediates the reduction of CoQ10 [13]. We thus sought to determine which plasma membrane ubiquinone reductase regenerates CoQ10H_2_ and functions as a radical-trapping antioxidant suppressing the propagation of lipid peroxides in melanoma cells. For this purpose, we evaluated FSP1 and NQO1 protein and mRNA levels in a panel of melanoma cell lines (Fig. 4A–C). FSP1 protein level was found to be significantly and similarly upregulated in all melanoma cell lines, highlighting its important role in melanoma survival. On the other hand, NQO1 protein level varied with significant upregulation only in RPMI-7951, SkMel24 and SkMel28 cell lines. We also compared FSP1 and NQO1 expression upon treatment with known inducers of different types of cell death: staurosporine (apoptosis), CH (lipid peroxidation) and RSL3, FIN56 and Erastin (ferroptosis) (Figs. S4A–B). UBIAD1 levels were also used as control (Fig. S4C). Interestingly, while FSP1 and UBIAD1 levels did not seem to be altered by these treatments, NQO1 expression was significantly stimulated by ferroptosis stimuli in SkMel28. These data suggested that NQO1 is differently regulated by different cell death conditions (apoptosis, ferroptosis or lipid peroxidation) making it an interesting target for further analyses.

To study the function of NQO1 in melanoma cells, we examined ROS and lipid peroxidation levels in SKMel28, A375 and Mel Juso cell after NQO1^KD^. NQO1 loss promotes increase of ROS levels similarly to UBIAD1^KD^ (Fig. S4D). NQO1 loss promotes also lipid peroxidation as evaluated by Bodipy C11 staining (Fig. 4D). We then performed a rescue experiment with idebenone and we found that idebenone treatment was indeed able to rescue lipid peroxidation after NQO1^KD^ in melanoma cells (Fig. 4E). This evidence further indicates that NQO1 enzyme counteracts lipid peroxidation and ROS generation in melanoma cells.

To further explore the role of NQO1 in melanoma cells-survival, we knock-downed NQO1 in SkMel28 and A375 melanoma cells and performed cell proliferation assays. NQO1^KD^ resulted in a significant reduction of cell viability of both SkMel28 and A375 cell lines (Fig. 4F). Also in this case, both cyclin A and RRM2 levels dropped in NQO1^KD^ conditions, confirming a block in cell proliferation (Fig. S4E). To demonstrate the existence of a UBIAD1/CoQ10/NQO1 axis that regulates and maintains cell survival in ROS-dependent melanoma cell lines, we sought to understand whether the two enzymes could have a synergistic effect. For this purpose, we carefully titered UBIAD1 and NQO1 knockdown in SkMel28 and A375 melanoma cell lines at suboptimal level (UBIAD1^KD Low^ and NQO1^KD Low^) (Fig. S4F) to avoid complete cell death as for UBIAD1^KD^ and NQO1^KD^ (Fig. 2A–B and Fig.4F). Next, we compared the effects of UBIAD1 and NQO1 silencing alone or in combination regarding cell viability and cell death (Fig. 4G). We found that a low level of UBIAD1 knockdown (UBIAD1^KD Low^) or NQO1 (NQO1^KD Low^) had less extreme effects on cell survival than those caused by a stronger KD (UBIAD1^KD^ or NQO1^KD^). However, the concomitant mild silencing of both UBIAD1 and NQO1 enzymes had a dramatic effect on cell survival (UBIAD1^KD Low^ + NQO1^KD Low^), suggesting a synergistic effect of the two enzymes in protecting melanoma cells from cell death.

Last, we evaluated the changes in mRNA expression of UBIAD1, NQO1 and FSP1 upon UBIAD1^KD^ or NQO1^KD^ in melanoma cells (Fig. 4H). Upon UBIAD1^KD^, both SkMel28 and A375 lines showed a slight downregulation of NQO1. Differently, mRNA level of FSP1 did not change in SkMel28 upon UBIAD1^KD^, but significantly increased in A375. This would probably mean that FSP1 could be engaged instead of NQO1 as CoQ10-reducing enzyme in this cell line, also considering the low level of NQO1 in A375 cells (Fig. 4B). NQO1^KD^, on the other hand, did not induce any changes neither in UBIAD1, nor in FSP1 in both cell lines. We conclude that NQO1 is located downstream of UBIAD1 and cooperates with it to protect BRAF-mutated melanoma cells from lipid peroxidation and cell death.

**Fig. 5:**
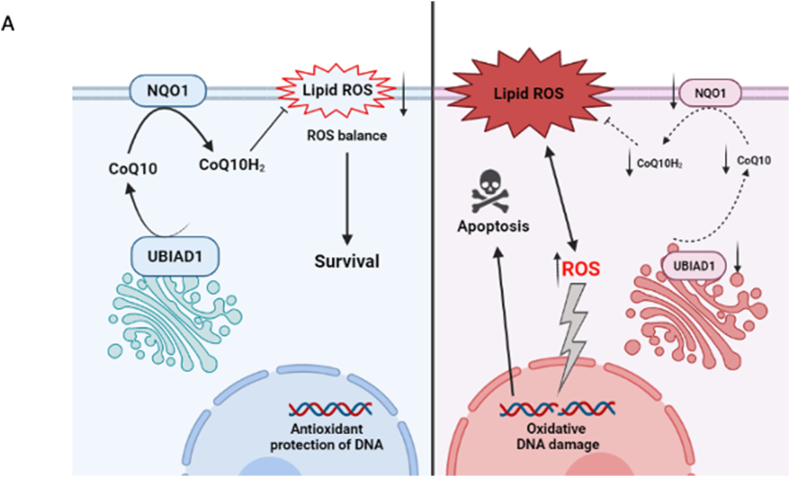
Schematic representation of UBIAD1-mediated CoQ10 protection against lipid peroxidation in melanoma cells. (A) Proposed mechanism of UBIAD1- and NQO1-mediated protection against lipid peroxidation in the normal situation (on the left): non-mitochondrial CoQ10 is synthesized by UBIAD1 in Golgi and transported to plasma membrane, where it is reduced by NQO1 to protect against lipid peroxidation (the scheme is designed using Biorender.com). In UBIAD1^KD^ and NQO1^KD^ cells (on the right) loss of reduced plasma membrane CoQ10 leads to lipid peroxidation, increased ROS levels and apoptotic cell death.

In summary, we suggest that NQO1 protein is responsible for CoQ10 redox regeneration downstream of the UBIAD1/CoQ10 axis in melanoma cell lines. When melanoma cells experience UBIAD1/CoQ10/NQO1 loss, ROS levels increase and promote lipid peroxidation as well as apoptotic cell death (Fig. 5A). The effect of UBIAD1/CoQ10 and NQO1 in survival of melanoma cell lines *in vitro* suggests to further consider these two enzymes as new therapeutic targets in melanoma research.

## 4 Discussion

Accumulating evidence indicates that melanoma cells overcome oxidative stress by developing different strategies [1,7]. The purpose of this study is to demonstrate that UBIAD1 is essential for melanoma survival by providing antioxidant protection through CoQ10 synthesis. UBIAD1 is a *trans*-membrane protein, localized in different cellular compartments and responsible for the biosynthesis of non-mitochondrial CoQ10 [22]. It was reported that UBIAD1 is involved in a variety of human diseases [[44], [45], [46]]. It also plays a tumor-suppressing role in bladder cancer [47,48]. Concerning melanoma, high UBIAD1 mRNA level is associated with poor prognosis (OS) in melanoma patients based on TCGA-Skin Cutaneous Melanoma datasets. To determine the functional role of UBIAD1 in melanoma progression we first tested mRNA and protein levels of UBIAD1 in a panel of melanoma cell lines and observed that UBIAD1 is significantly upregulated in melanoma cells with respect to melanocytes. By immunofluorescence we showed that UBIAD1 is co-localized mainly to Golgi and to a lesser extent to ER in melanoma cells with respect to melanocytes, consistently with previous data suggesting that the biosynthesis of non-mitochondrial CoQ10 by UBIAD1 is localized to Golgi [22,49]. We selected some BRAF- and NRAS-mutated cell lines such as SkMel28, A375 and Mel Juso to study the role of UBIAD1 in the antioxidant defense of melanoma.

In line with previous studies [22,50,51], we then showed that UBIAD1^KD^-dependent depletion of plasma membrane CoQ10 blocks cell proliferation and triggers ROS increase, lipid peroxidation and apoptotic cell death in melanoma cells. Importantly, redox lipid balance, DNA damage and viability of UBIAD1^KD^ cells were rescued by restoring the antioxidant potential of plasma membranes through idebenone treatment.

Accumulating evidence indicated that UBIAD1 is tightly linked to cholesterol metabolism as some studies show that UBIAD1 suppresses intracellular cholesterol metabolism [45] and other studies report that UBIAD1 inhibits HMGCR degradation by direct interaction, thus elevating the cholesterol level [30]. Our mass-spectrometry analysis of different lipid metabolites did not display any significant difference between control and UBIAD1^KD^ samples regarding cholesterol and TAG, except a slight increase of cholesterol esters in A375 cell line upon UBIAD1^KD^. While it was reported that UBIAD1 is responsible for the biosynthesis of vitamin K2 [52], we did not detect vitamin K2 in melanoma cells by HPLC measurement (data not shown), confirming that UBIAD1 is not required for generating Vitamin K2 in melanoma cells. Together, our results suggest that UBIAD1 serves as an antioxidant defense through the biosynthesis of non-mitochondrial CoQ10, but not by the regulation of cholesterol metabolism.

We then explored the dependency of melanoma cells on CoQ10 downstream of its synthesis. CoQ10 can be reduced by different enzymes in cancer cells, for example FSP1 in the plasma membrane [13] and DHODH in the mitochondrial inner membrane [14]. We hypothesized that in melanoma cells also NQO1, transcriptional target of NRF2, could be responsible for the regeneration of the reduced form of CoQ10, since NQO1 is part of a plasma membrane redox system and elevated levels of NQO1 are associated with poor melanoma patient outcome [15,41,53]. According to our hypothesis, we found that NQO1^KD^ induced ROS increase and lipid peroxidation and that NQO1 is required for survival in melanoma cells. Such defects resemble UBIAD1^KD^ conditions. Finally, we discovered that the simultaneous but mild knockdown of UBIAD1 and NQO1 have a synergistic effect with cell death levels comparable with the full KD of only one enzyme.

Recently, it was proposed a new model for antioxidant enzymes involved in defense against lipid peroxidation in cancer: GPx4 in the cytosol and mitochondria, FSP1 on the plasma membrane, and DHODH in mitochondria [14]. However, we suggest that the mechanism of antioxidant protection is more complicated and composed of more different antioxidant enzymes. The contribution of each enzyme is “context-dependent” on the chosen model of cancer, as the regulation of each enzyme is dependent on the ROS-modulators. Another important point is that cancer cells maintain high levels of ROS, very close to a death threshold. Thus, the impairment of even only one antioxidant pathway can trigger cell death. This view is supported by our findings that UBIAD1/CoQ10/NQO1 axis disruption is sufficient to trigger cell death in melanoma cell lines despite the presence of other antioxidant enzymes. However, it still remains to be further explored the functional cross-talk between UBIAD1 and NQO1 in melanoma progression and whether the blocking of these enzymes would represent a valid therapeutic approach to treat melanoma.

## 5 Conclusion

In this study, we showed that UBIAD1, a transmembrane enzyme localized in the Golgi apparatus, plays a key role in suppressing lipid peroxidation and promoting melanoma survival through CoQ10 synthesis and NQO1-dependent plasma membrane redox regulation. We envision UBIAD1 and NQO1 blockade as novel therapeutic strategies in melanoma. In particular, it could be interesting to study UBIAD1/CoQ10/NQO1 axis in those situations where oxidative stress drives resistance to therapy (e.g. BRAF and MEK inhibitors-resistant melanoma) [54,55].

## Author contributions

LA and MMS conceived the concept of the study. MMS provided supervision and wrote the manuscript. MMS, LA, GT and MR were involved in the experimental design and writing. MS, PC performed the metabolomics analyses. SP, FAT performed bioinformatic analyses on TCGA dataset. LA, GT carried out biological activity assays. LA, GT and MR performed cell culture experiments. LA, GT, MR, MS, PC and MMS interpreted the data. All authors agreed on the final version of the manuscript. All authors commented on the manuscript.

The authors declare no competing interests.

## Appendix A Supplementary data

Figure S1.UBIAD1 and *COQ2* expression in melanoma cell lines.(A) Western blot analysis of UBIAD1 protein level in human melanocytes (NHEM, HEMa-LP), human epidermal keratinocyte (HaCaT) and melanoma cell lines. β-actin was used as loading control.(B) qRT-PCR quantifications of C*OQ2* mRNA levels in a panel of melanoma cell lines. *COQ2* mRNA level was normalized to β-actin and reported as relative to HEMa-LP (set at 1). 1-way ANOVA with Dunnett‘s multiple comparisons test (HEMa-LP as a control) was used to quantify statistical significance. Error bars represent SEM, n ≥ 3.

Figure S2. UBIAD1 loss does not affect specific signaling pathways or global lipid metabolism in melanoma cells.(A) Western blot analysis and (B) corresponding quantification of NF-KB p65, AMPK, ERK1/2, AKT and mTOR/p6 signaling pathways, involved in melanoma survival, upon UBIAD1^KD^ in SkMel28 cells.(C) Western blot analysis and (D) corresponding quantification of NF-KB p65, AMPK, ERK1/2, AKT and mTOR/p6 signaling pathways, involved in melanoma survival, upon UBIAD1^KD^ in A375 cells.(E) Western blot analysis and (F) corresponding quantification of NF-KB p65, AMPK, ERK1/2, AKT and mTOR/p6 signaling pathways, involved in melanoma survival, upon UBIAD1^KD^ in Mel Juso cells.(G) Representative images of FACS assay of DHE staining in SkMel28 and A375 upon UBIAD1^KD Low^ or UBIAD1^KD^. Scatter plot is shown with viability (FVS 780) on the y axis and DHE on the x axis. DHE-positive cells are colored in red. The horizontal line divides “Dead cells” (above) from “Alive cells” (below). Menadione was used as positive control.(H) Representative images of FACS assay of DCFH-DA staining in SkMel28 and A375 upon UBIAD1^KD Low^ or UBIAD1^KD^. Scatter plot is shown with viability (FVS 780) on the y axis and DCFH-DA on the x axis. DCFH-DA -positive cells are colored in red. The horizontal line divides “Dead cells” (above) from “Alive cells” (below). H_2_O_2_ was used as positive control.(I) HPLC-MS analyses of different types of lipids upon UBIAD1^KD^: triacylglycerols (TAG), cholesterol esters (CE), free cholesterol (CO), total cholesterol (CT). Unpaired Student two-tailed *t*-test was used to quantify statistical significance. Error bars represent SEM, n = 6.

Figure S4. NQO1 contributes to CoQ10 redox homeostasis in melanoma cells.(A-C) Western blot representative images of FSP1 (A), NQO1 (B) and UBIAD1 (C) protein levels in melanoma cells upon induction of cell death by different treatments. 1-sample *t*-test was used to quantify statistical significance. Error bars represent SEM, n = 3.(D) FACS assay of DHE and DCFH-DA staining in SkMel28 and A375 upon NQO1^KD^. Data are shown as percentage of DHE- or DCFH-DA-positive cells over singlets. Unpaired Student two-tailed *t*-test was used to quantify statistical significance. Error bars represent SEM, n ≥ 3.(E) Western blot analysis of cyclin A and RRM2 proteins upon NQO1^KD^ in SkMel28 and A375 melanoma cell lines.(F) qRT-PCR quantifications of *UBIAD1* and *NQO1* mRNA levels upon silencing using low or high lentivirus loads. Unpaired two-tailed t-tests were used to quantify statistical significance. Data points represent technical replicates, error bars represent SEM, n = 3.
