# Supplementary figures and images for: UBIAD1 and CoQ10 protect melanoma cells from lipid peroxidation-mediated cell death
Source: Redox Biol. 2022 Feb 18;51:102272. doi: 10.1016/j.redox.2022.102272 (PMC8902599; doi:10.1016/j.redox.2022.102272)

A

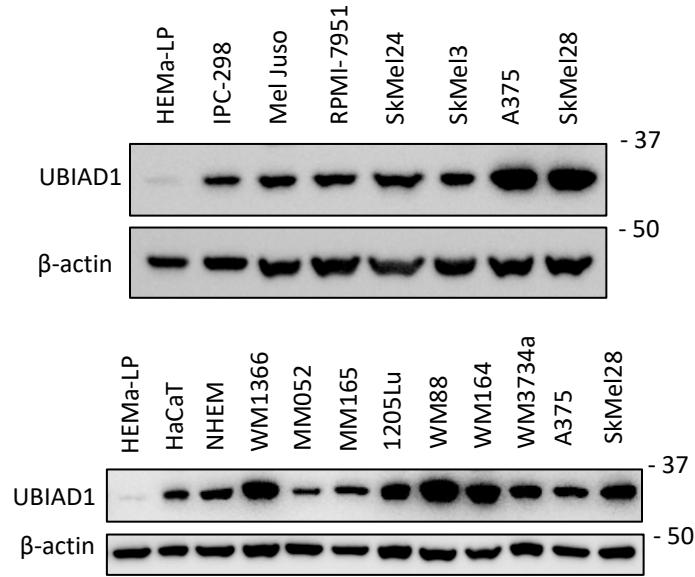

B

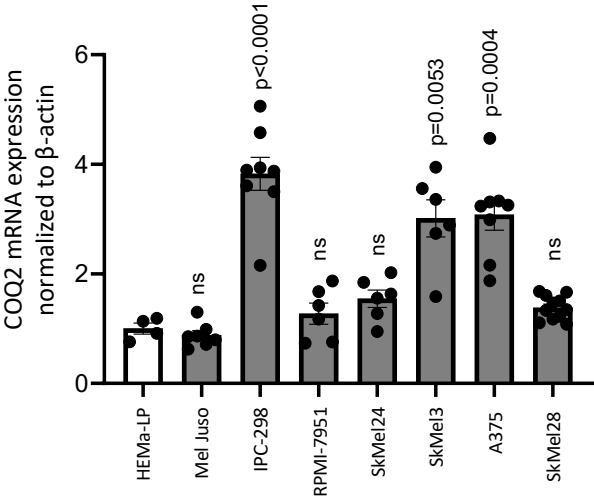

Figure S1

A

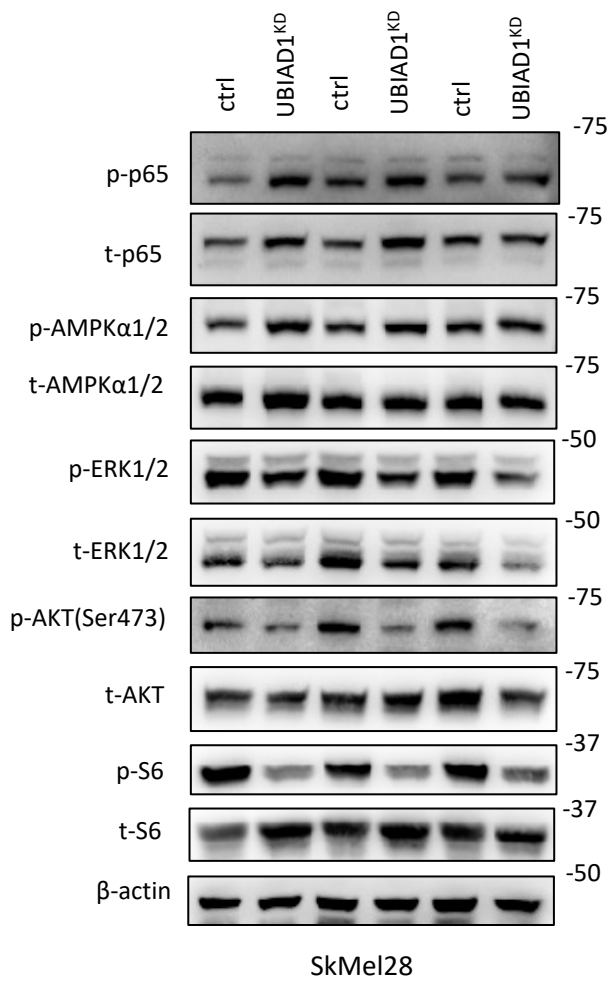

B

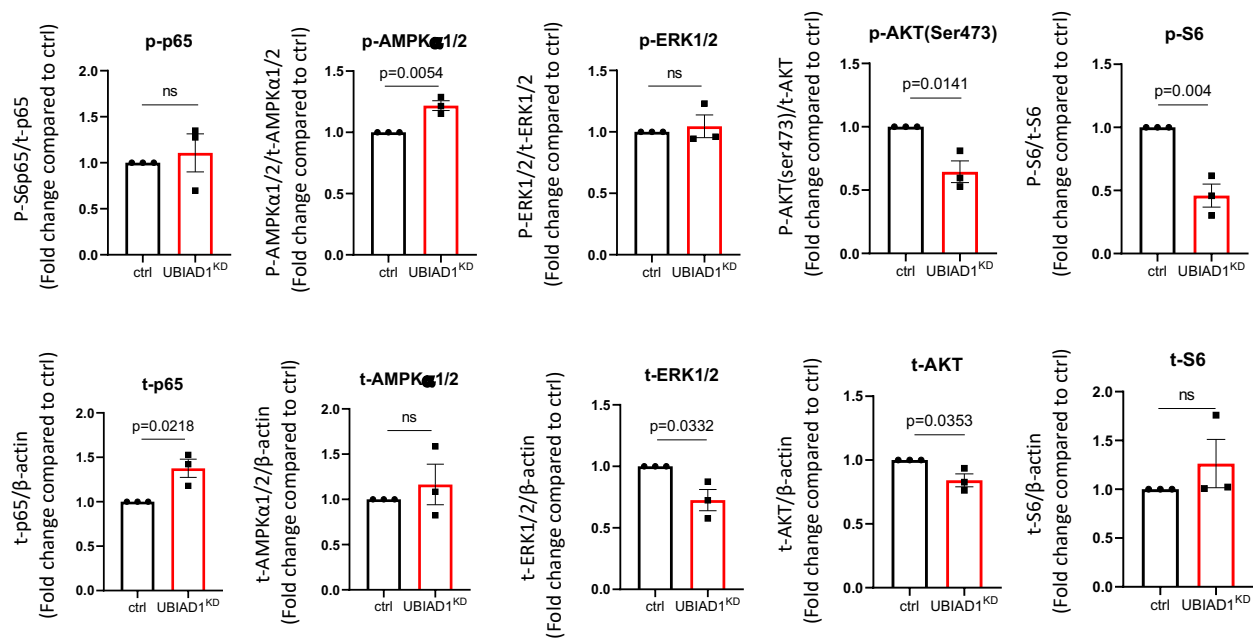

Figure S2

C

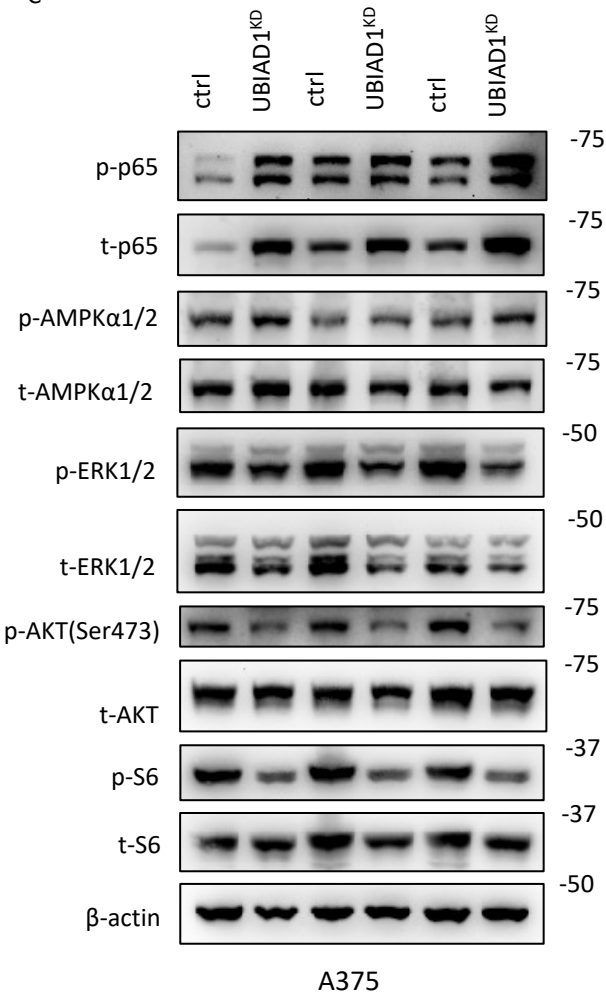

D

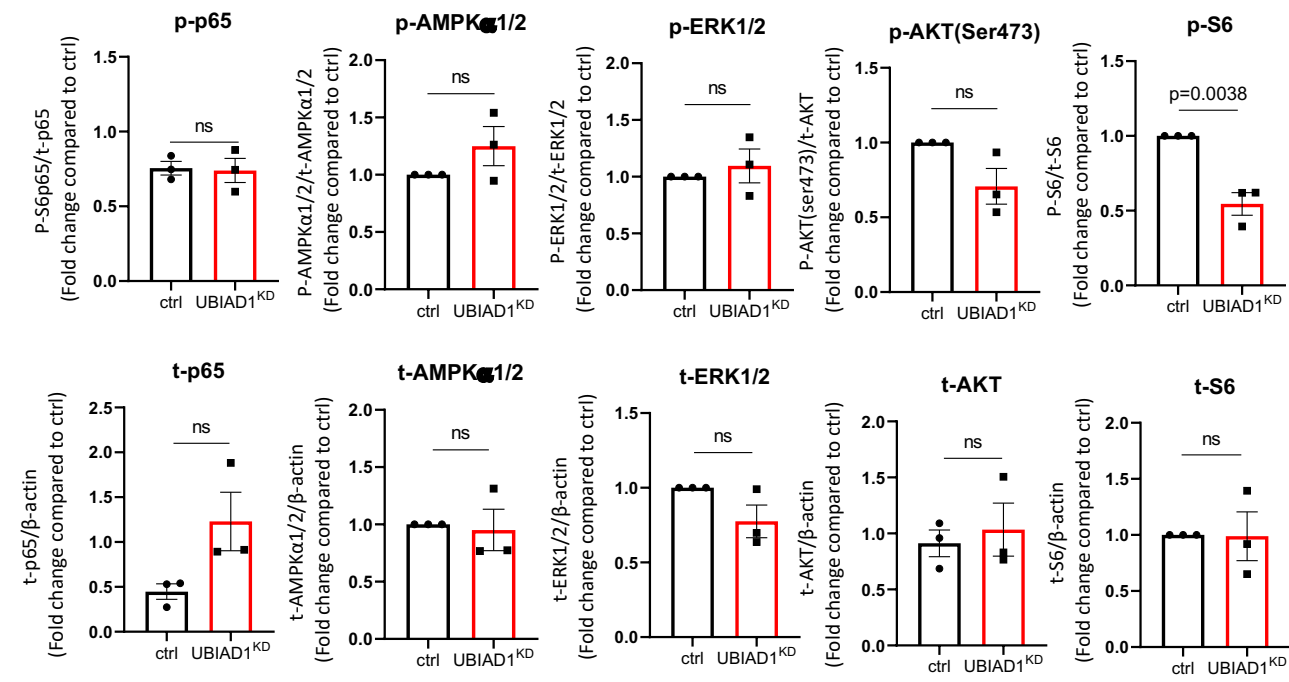

Figure S2

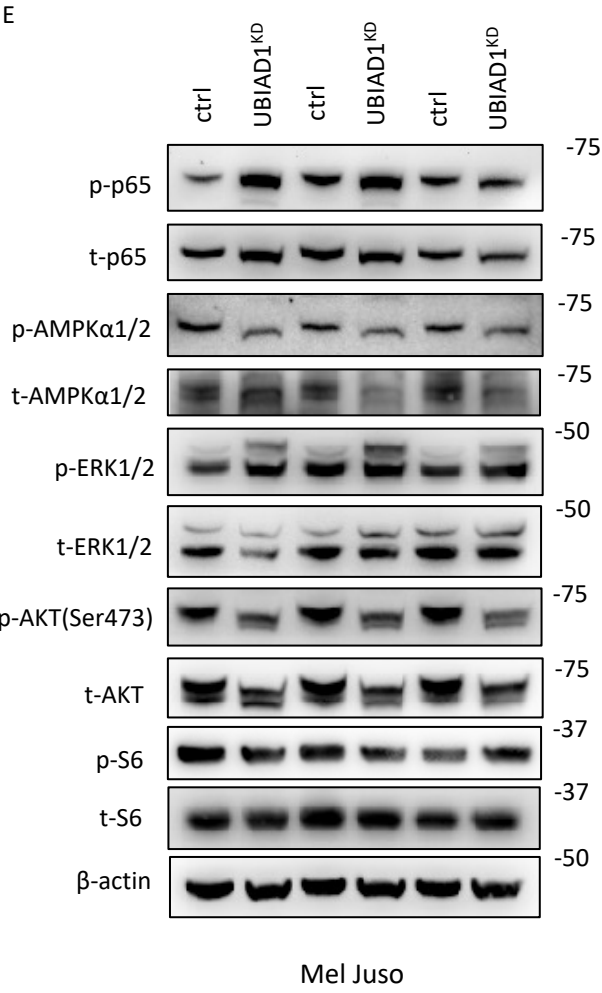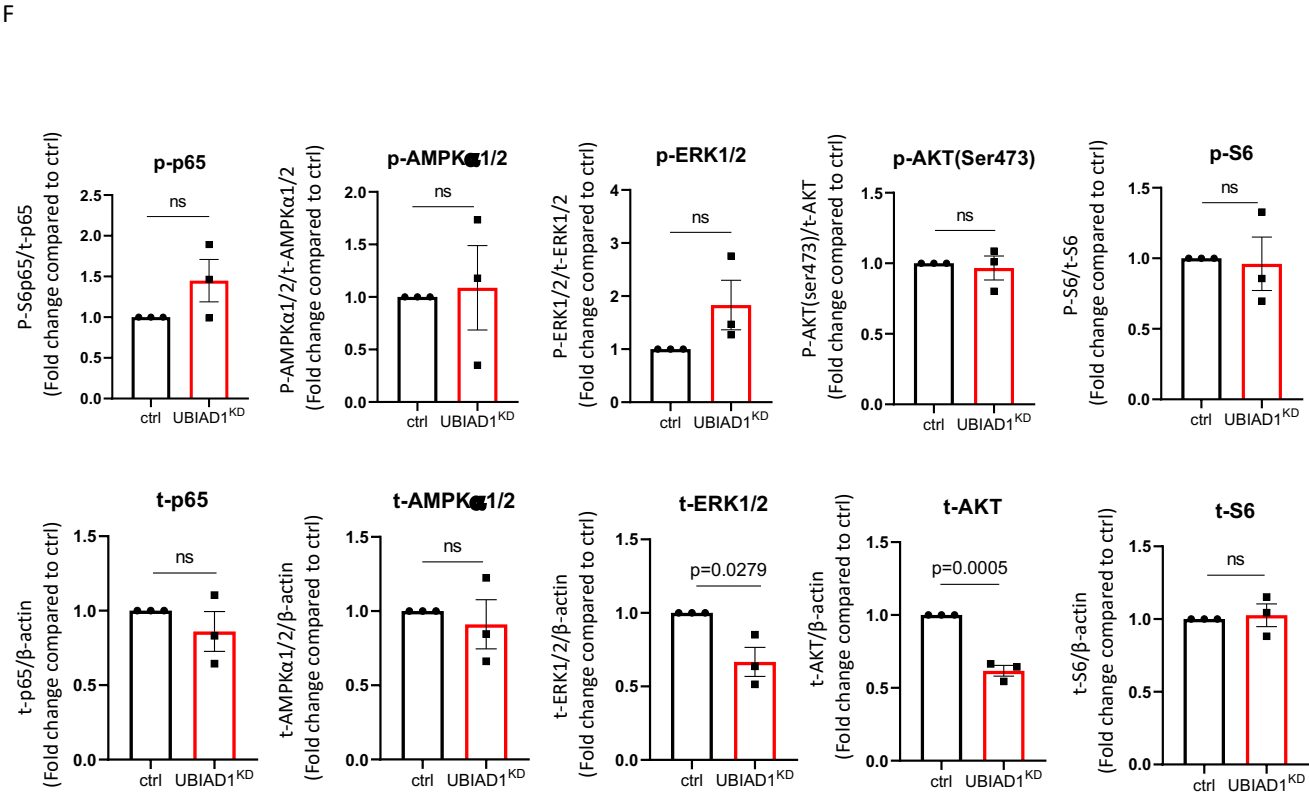

Figure S2

G

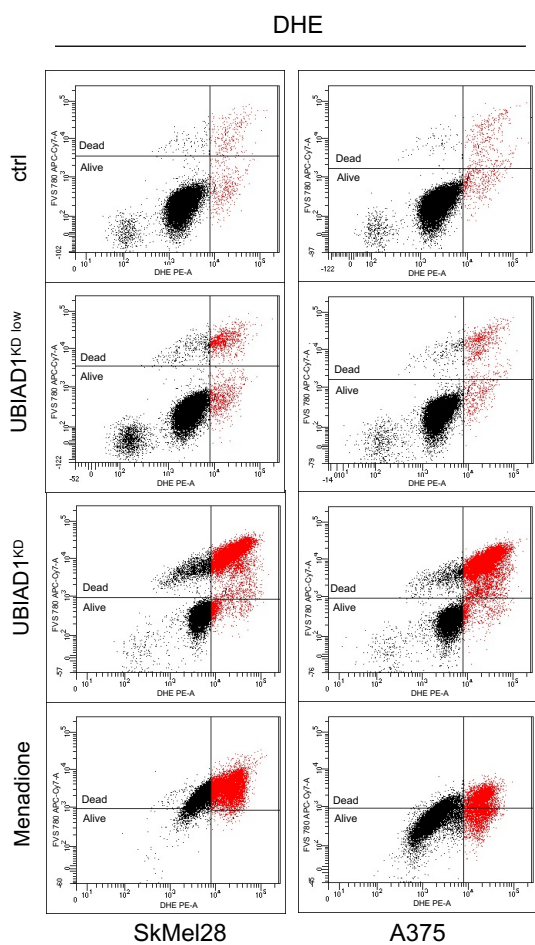

H

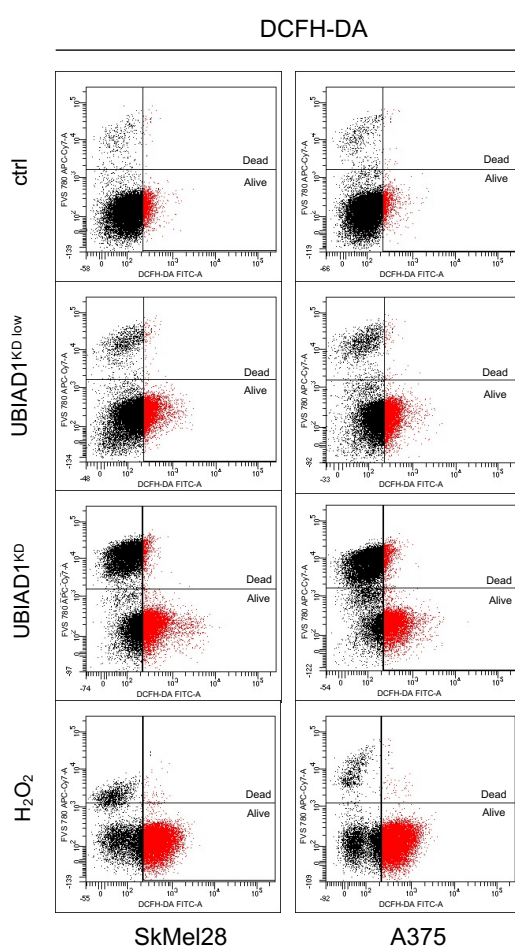

I

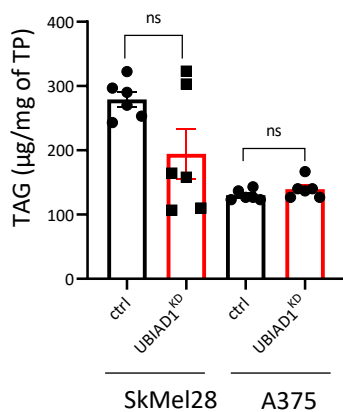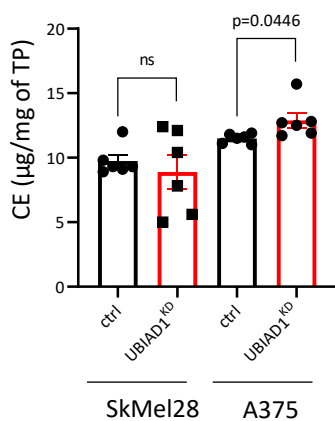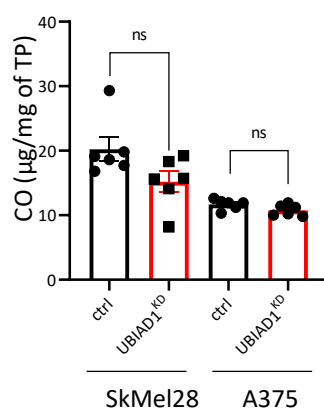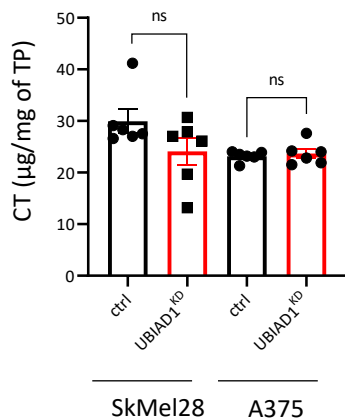

Figure S2

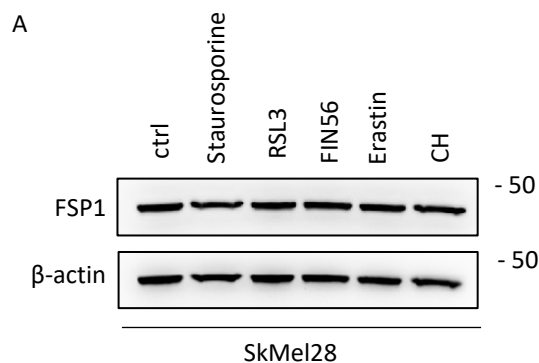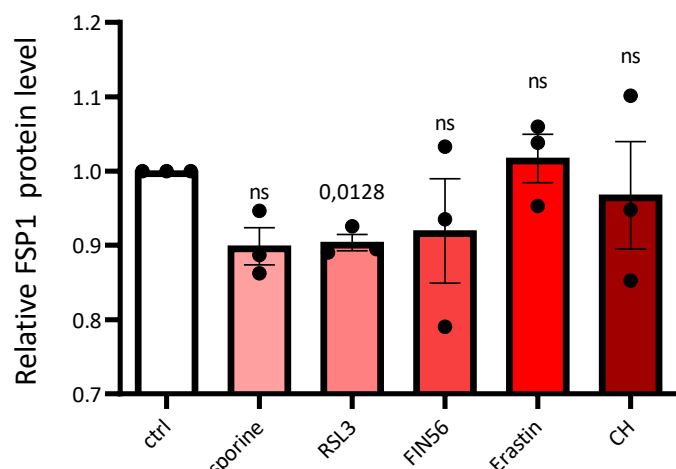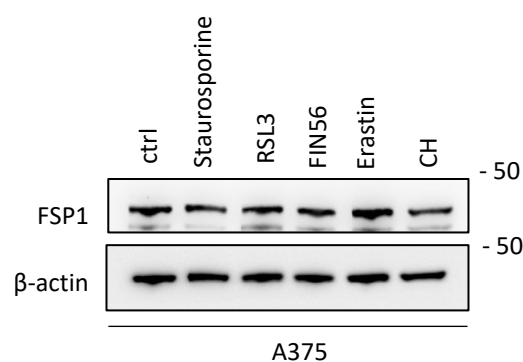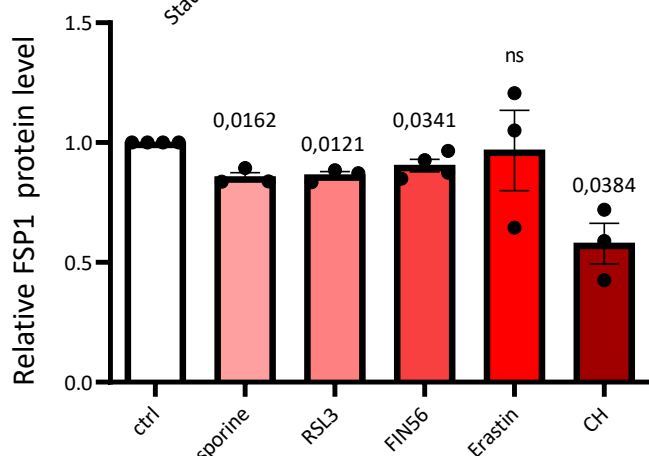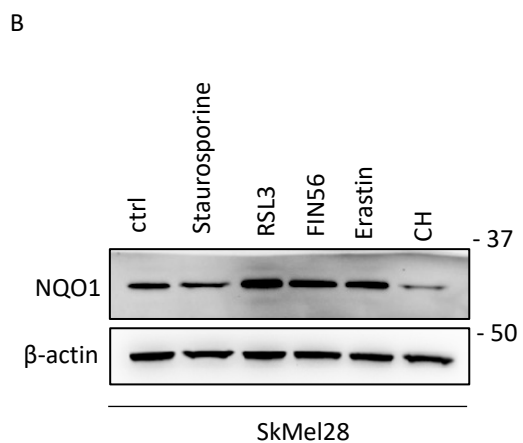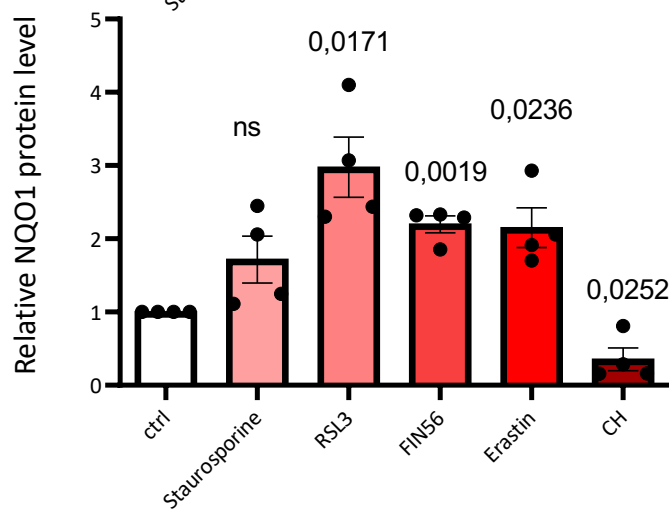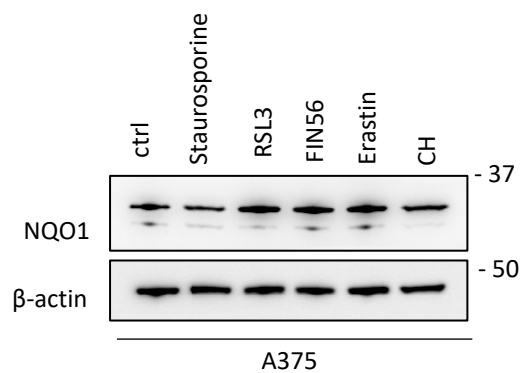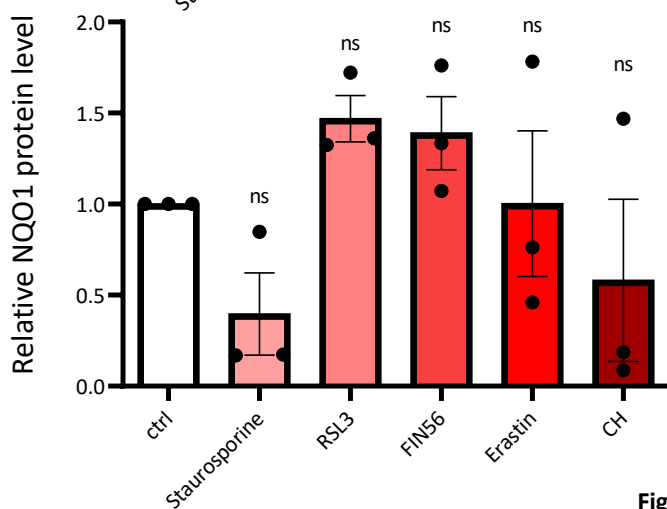

Figure S4

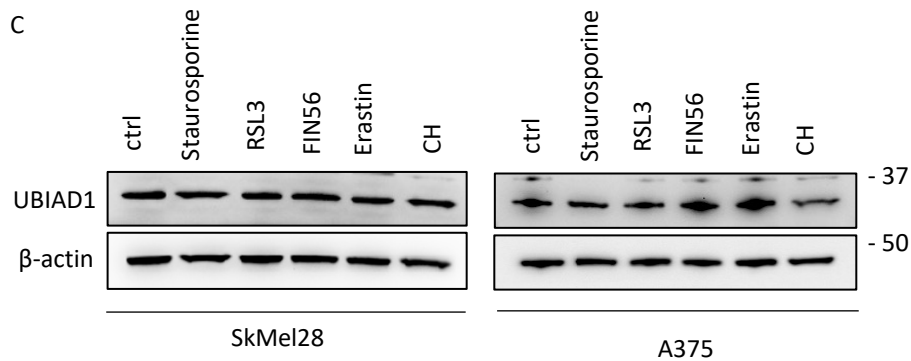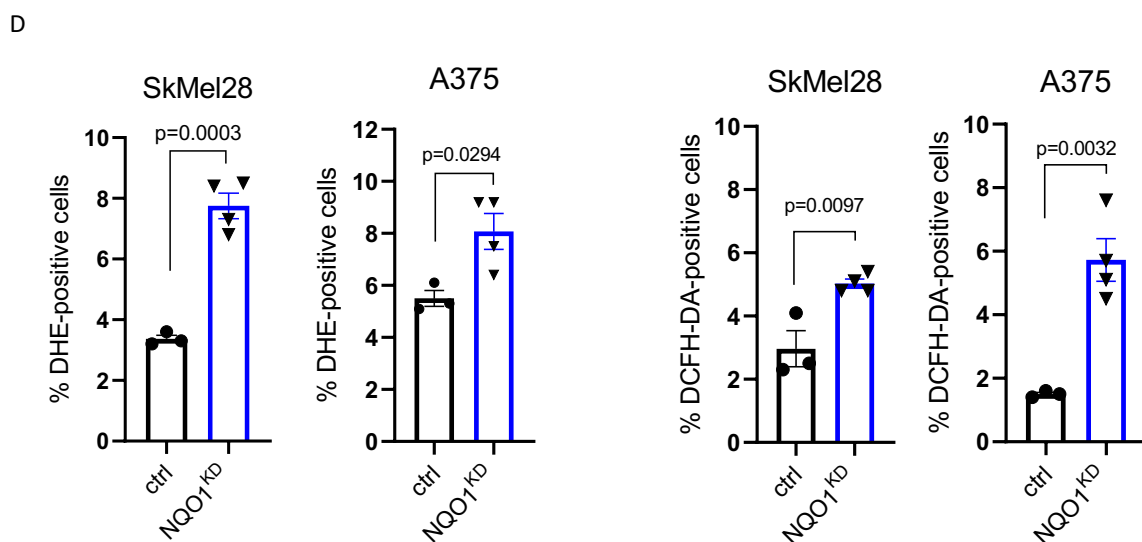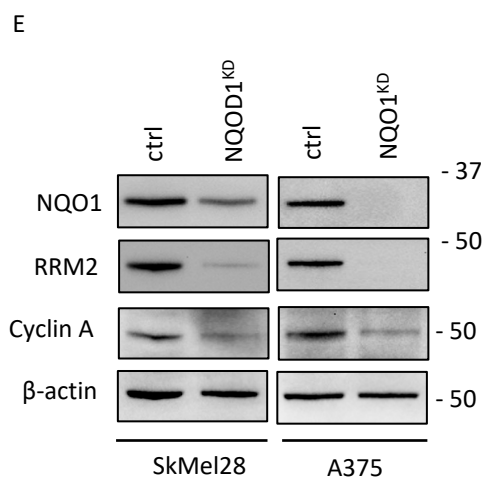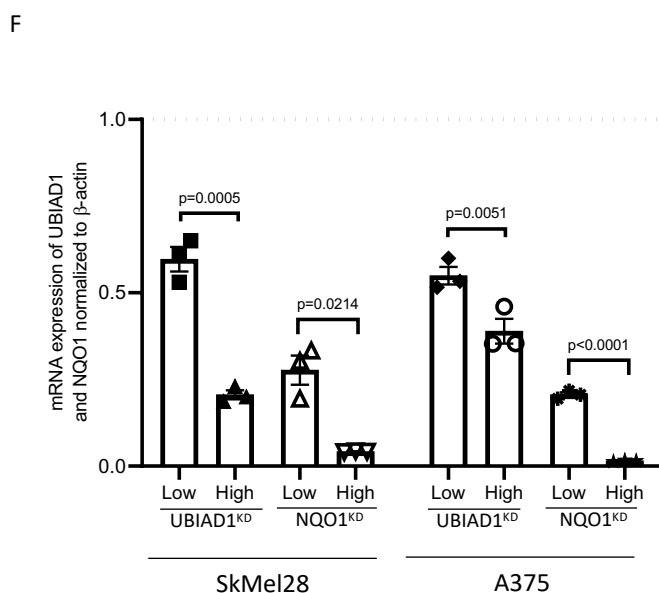

Figure S4

Supplement: Multimedia component 1 [file mmc1.pdf]
